# Simulated binding of transcription factors to active and inactive regions folds human chromosomes into loops, rosettes and topological domains

**DOI:** 10.1093/nar/gkw135

**Published:** 2016-04-08

**Authors:** Chris A. Brackley, James Johnson, Steven Kelly, Peter R. Cook, Davide Marenduzzo

**Affiliations:** 1 SUPA, School of Physics & Astronomy, University of Edinburgh, Peter Guthrie Tait Road, Edinburgh, EH9 3FD, UK; 2 Department of Plant Sciences, University of Oxford, South Parks Road, Oxford OX1 3RB, UK; 3 Sir William Dunn School of Pathology, University of Oxford, South Parks Road, Oxford, OX1 3RE, UK

## Abstract

Biophysicists are modeling conformations of interphase chromosomes, often basing the strengths of interactions between segments distant on the genetic map on contact frequencies determined experimentally. Here, instead, we develop a fitting-free, minimal model: bivalent or multivalent red and green ‘transcription factors’ bind to cognate sites in strings of beads (‘chromatin’) to form molecular bridges stabilizing loops. In the absence of additional explicit forces, molecular dynamic simulations reveal that bound factors spontaneously cluster—red with red, green with green, but rarely red with green—to give structures reminiscent of transcription factories. Binding of just two transcription factors (or proteins) to active and inactive regions of human chromosomes yields rosettes, topological domains and contact maps much like those seen experimentally. This emergent ‘bridging-induced attraction’ proves to be a robust, simple and generic force able to organize interphase chromosomes at all scales.

## INTRODUCTION

The conformations adopted by human chromosomes in 3D nuclear space are currently an important focus in genome biology, as they underlie gene activity in health, aging and disease (1). Chromosome conformation capture (3C) and high-throughput derivatives like ‘Hi-C’ allow contacts between different chromatin segments to be mapped (2). Inspection of the resulting contact maps reveals some general principles, including the following ones. (i) Each chromosome folds into distinct ‘topological domains’ during interphase (but not mitosis when transcription ceases), domains contain 0.1–2 Mbp, active and inactive regions tend to form separate domains, and sequences within a domain contact each other more often than those in different domains (2–8). (ii) Domains seem to be specified locally, as the same 20-Mbp region in a chromosomal fragment or in the intact chromosome make much the same contacts (7). (iii) Bound transcription factors like CTCF (the CCCTC-binding factor) and active transcription units are enriched at domain ‘boundaries’ (3,7). (iv) Factors bound to promoters and enhancers stabilize loops (7,9–14). (v) Co-regulated genes utilizing the same factors often contact each other when transcribed (10,15–18). (vi) Single-cell analyses show no two cells in the same population share exactly the same contacts, but the organization is non-random as certain contacts are seen more often than others (19). (vii) This organization is conserved; in budding yeast (20) and *Caulobacter crescentus* (21), ‘chromosomal interaction domains’ are separated by strong promoters and the bacterial ones are eliminated by inhibiting transcription. These principles point to central roles for transcription orchestrating this organization, with transcription factors providing the required specificity.

Biophysicists are attempting to model this organization (2,6,19,21–45), often basing the strength of interactions between segments distant in 1D sequence space on contact frequencies determined using Hi-C (2,19,25,28–29,36,40–45). To understand the principles underlying the organization, we use a minimal model without such fitting. This model was originally developed to analyze non-specific binding of proteins like histones to DNA (32,39); here, we adapt it to include specific binding. Thus, spheres (representing transcription factors) bind briefly to cognate sites in strings of beads (representing chromatin) before dissociating. These factors provide an obvious connection with transcription, as they often associate with RNA polymerase (which can remain tightly bound to the template for ∼10 min as it transcribes the average human gene—a binding that is also specific in the sense it occurs throughout a transcription unit but not elsewhere). [However, here, we only model transient binding.] Like many transcription factors (or complexes made up of several of these factors), ours are ‘bivalent’ (or ‘multivalent’); they can bind simultaneously to two or more segments of one fiber, to create molecular ‘bridges’ that stabilize loops. More generally, our spheres could represent any multivalent DNA-binding complex that binds specifically. Our model is similar to the strings and binders switch (SBS) model introduced by Nicodemi *et al*. (24,30) with some notable differences: (i) both the polymer and the multivalent factors can occupy any position in 3D space in our model, whereas in the SBS model they can only be found at nodes in a 3D lattice, (ii) we use coarse grained molecular dynamics (MD) simulations instead of a Monte-Carlo approach and (iii) most importantly, our goal is different—we aim to directly and quantitatively compare contacts detected in simulations with those seen in Hi-C contact maps.

In contrast to previous models used to reach the same goal, our model is fitting free. Instead of beginning with experimentally-determined Hi-C data, we start with 1D information (i.e. whether a particular genomic region is transcriptionally active or not) and use it to generate a population of possible chromosome structures (considering fibers with more subunits than those used previously); only then, do we compare the resulting contacts with those seen experimentally. Remarkably, our coarse-grained MD simulations show fibers spontaneously fold into structures possessing the key features outlined above. We uncover an emergent force that can act through the binding of just two (or more) types of transcription factor to their cognate sites that is able to organize interphase chromosomes locally and globally—all without inclusion of any explicit attractive force between distant segments, or between factors.

## MATERIALS AND METHODS

MD simulations were run with the LAMMPS (Large-scale Atomic/Molecular Massively Parallel Simulator) code (46), run in Brownian dynamics (BD) mode (i.e., with a stochastic thermostat, see Supporting Data for more details). Chromatin fibers are modeled as bead-and-spring polymers using FENE bonds (maximum extension 1.6 times bead diameter, σ) and a bending potential that allows persistence length to be set. Protein:protein and chromatin:chromatin interactions involve only steric repulsion, and those between proteins and their binding sites are truncated and shifted Lennard–Jones interactions. All beads are confined within a cube with periodic boundary conditions. Additional details are listed in figure legends and/or Supplementary Data.

Simulations of human chromosomes included two kinds of factors/proteins, one binding to active euchromatin and the other to inactive heterochromatin. Chromatin beads were colored pink or light-green if interacting strongly or weakly, respectively, with red factors (representing factors and polymerases), gray if interacting with black proteins (representing HP1α) and blue if non-interacting. The Broad ChromHMM track (47) on the hg19 assembly of the UCSC Genome browser was used to determine pink/light green color: if a region of 90 bp or more within one bead is labeled as an ‘Active Promoter’ or ‘Strong Enhancer’ (states 1,4,5) then the whole bead is colored pink and similarly if a bead contains 90 bp or more of ‘Transcriptional Transition’ or ‘Transcriptional Elongation’ (states 9,10) it is colored light green. [The ChromHMM track was mapped by using a Hidden Markov Model (HMM), see (47) for details.] Data for GC content (Figures 4, 5 and Supplementary Figure S12D) or from the ChromHMM track (Supplementary Figure S12A and B) were used to determine gray color: (i) if GC content of the DNA contained in a chromatin bead was below the threshold specified in each figure legend, or (ii) if a region of 90 bp or more within one bead is labeled ‘Heterochromatin; low signal’ (state 13). These conventions allow one bead to have multiple colors. Figure legends give numbers of differently-colored beads in a simulation and affinities. Simulations included red factors (*n* = 300 in Figure 4 and Supplementary Figure S12; *n* = 400 in Figure 5), and black protein (*n* = 3000 in Figure 4 and Supplementary Figure S12; *n* = 4000 in Figure 5); for simplicity, protein size is equal to σ and interaction range to 1.8 σ.

**Figure 1.**
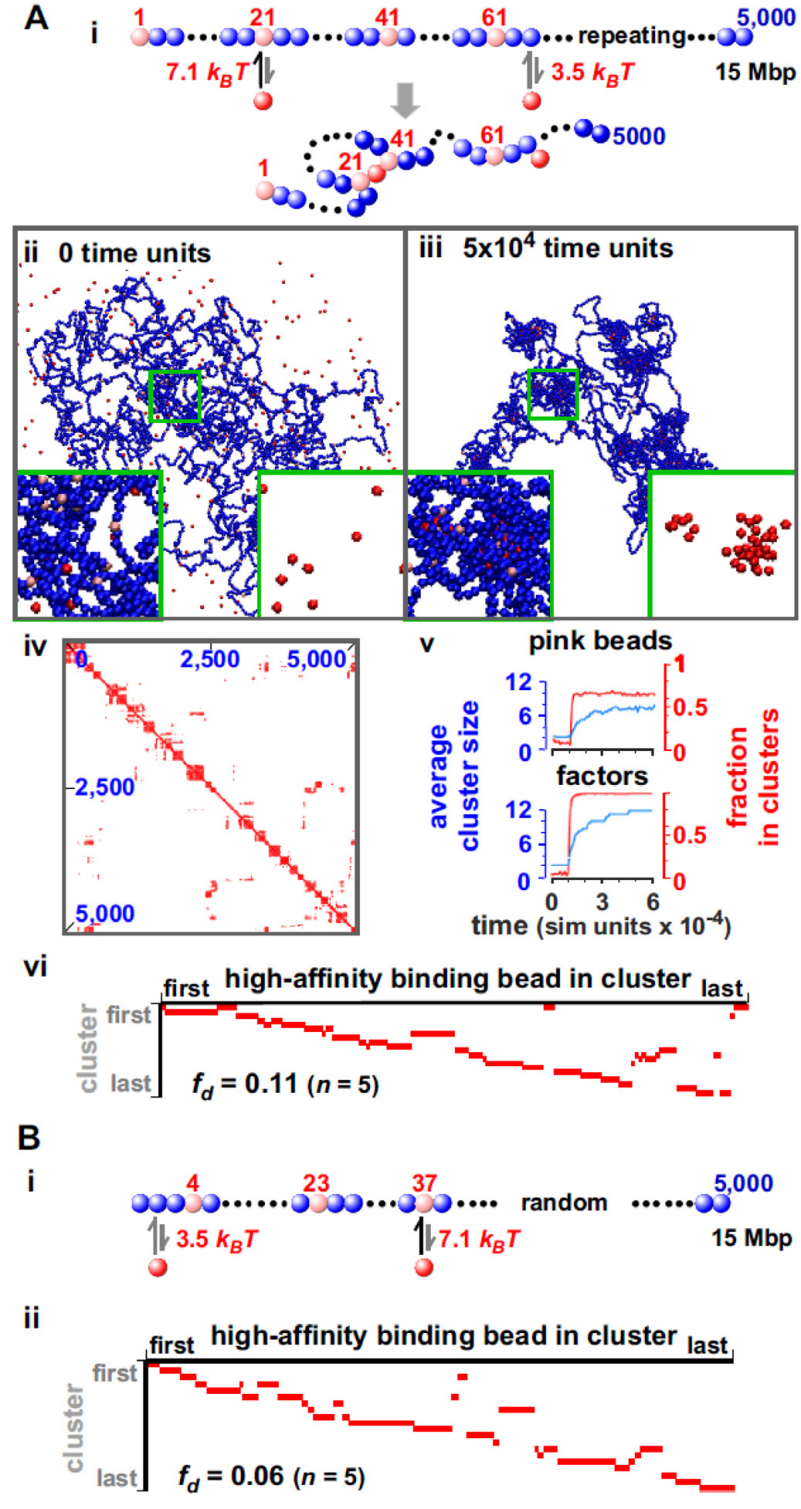
Bound ‘factors’ spontaneously cluster. (**A**) Regularly-spaced high-affinity binding sites. (i) Overview. MD simulations involved a 3-μm cube containing 250 30-nm red spheres (‘transcription factors’; volume fraction 0.01% or 15 nM), and a fiber of 5000 30-nm beads (15-Mbp ‘chromatin’, so each bead contains 3 kbp; persistence length 90 nm; volume fraction 0.26%, so chromatin is ‘dilute’). One bead in 20 is pink (with regular spacing), others are blue. Beads begin to interact (strength indicated) with factors after 10^4^ time units if centers lie within 54 nm; here, binding of a factor to beads 21 and 41 creates a loop. (ii,iii) Snapshots after different times; insets show magnifications of boxed areas (with/without chromatin). (iv) Contact map after 5 × 10^4^ time units (axes give bead numbers; data from one run). Here (and later, unless stated otherwise), a contact is scored if bead centers lie within 150 nm, and contacts made by 40 adjacent beads are binned; a red pixel then marks contacts between beads at positions indicated, with intensity (white to red) reflecting contact number (low to high). Blocks along the diagonal mark many contacts made by clusters of bound factors. (v) Average cluster size, and fraction in clusters, for pink beads and factors (data sampled every 1000 time units). Two or more pink beads are in one cluster if centers lie <90 nm apart. Small clusters form quickly, and slowly enlarge to the steady-state size. (vi) Rosettogram. A red pixel marks the presence of a high-affinity bead in a cluster; increasing numbers of abutting pixels in one row reflect increasing numbers of loops in a rosette involving near-neighbor high-affinity sites. Most clusters contain ≥2 loops. *f*_*d*_: disorganized fraction (average of 5 runs). (**B**) Randomly-distributed high-affinity binding sites. (i) Pink beads are randomly distributed over the fiber, with the same average linear density as in (A). (ii) Rosettogram. The structure is slightly more rosette-like than the one in (A) (reflected by a lower *f*_*d*_).

**Figure 2.**
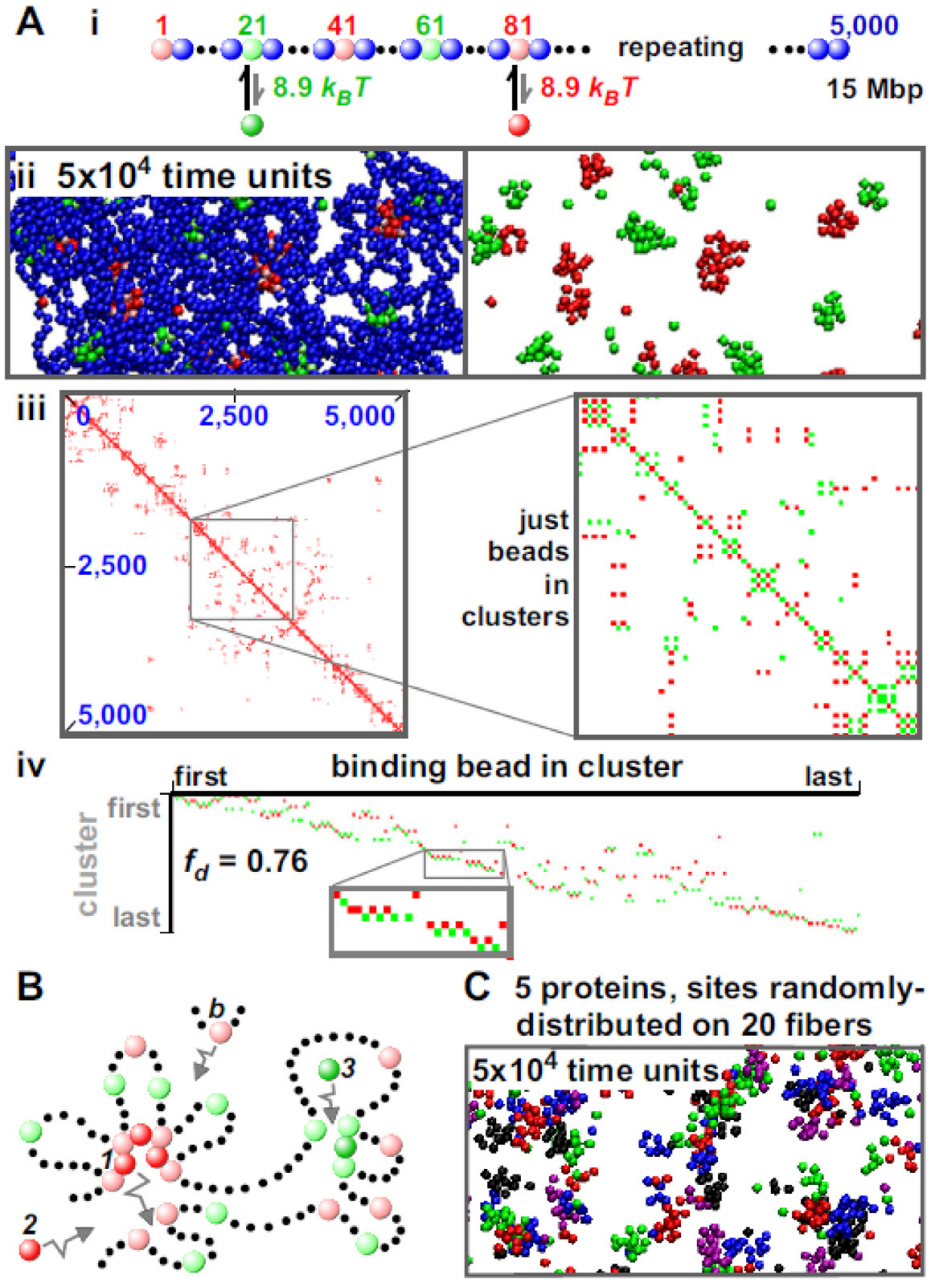
Self-assembly into ‘specialized’ clusters. MD simulations were run as in Figure 1, except for differences indicated. (**A**) Red (*n* = 250) and green (*n* = 250) factors interact with pink and light-green beads, respectively. (i) One bead in 20 is a binding bead (with regular spacing); the colors of binding beads alternate as indicated. (ii) Final snapshot of central region (with/without chromatin); clusters contain either red or green factors. (iii) Final contact map; blocks along the diagonal are small. The zoom shows a high-resolution map involving only binding beads in clusters; contacts are scored (without binning) if bead centers lie 90 nm apart (not 150 nm), and any binding beads are treated as if they possess the color of factor binding them. Here, red and green pixels mark contacts between two pink beads, or between two light-green beads: notably, there are no mixed contacts between a light-green and pink bead (these are shown in yellow in Supplementary Figure S3). Similarly-colored pixels rarely abut in a row, as the fiber passes back and forth between differently-colored clusters. (iv) Final rosettogram (pixels correspond to binding beads, and are colored as in the contact map zoom); rows rarely contain abutting pixels of one color (reflected by a high *f*_*d*_). (**B**) How ‘specialized’ clusters form. See text. (**C**) Red, green, dark-blue, purple and black factors (500 of each) bind (7.1 *k*_*B*_*T*) to five sets of cognate sites scattered randomly along 20 identical fibers (each with 2000 beads representing 6 Mbp). The snapshot (taken after 5 × 10^4^ units; DNA not shown for clarity) shows that each factor tends to cluster with similarly-colored ones. See also Supplementary Figure S5.

**Figure 3.**
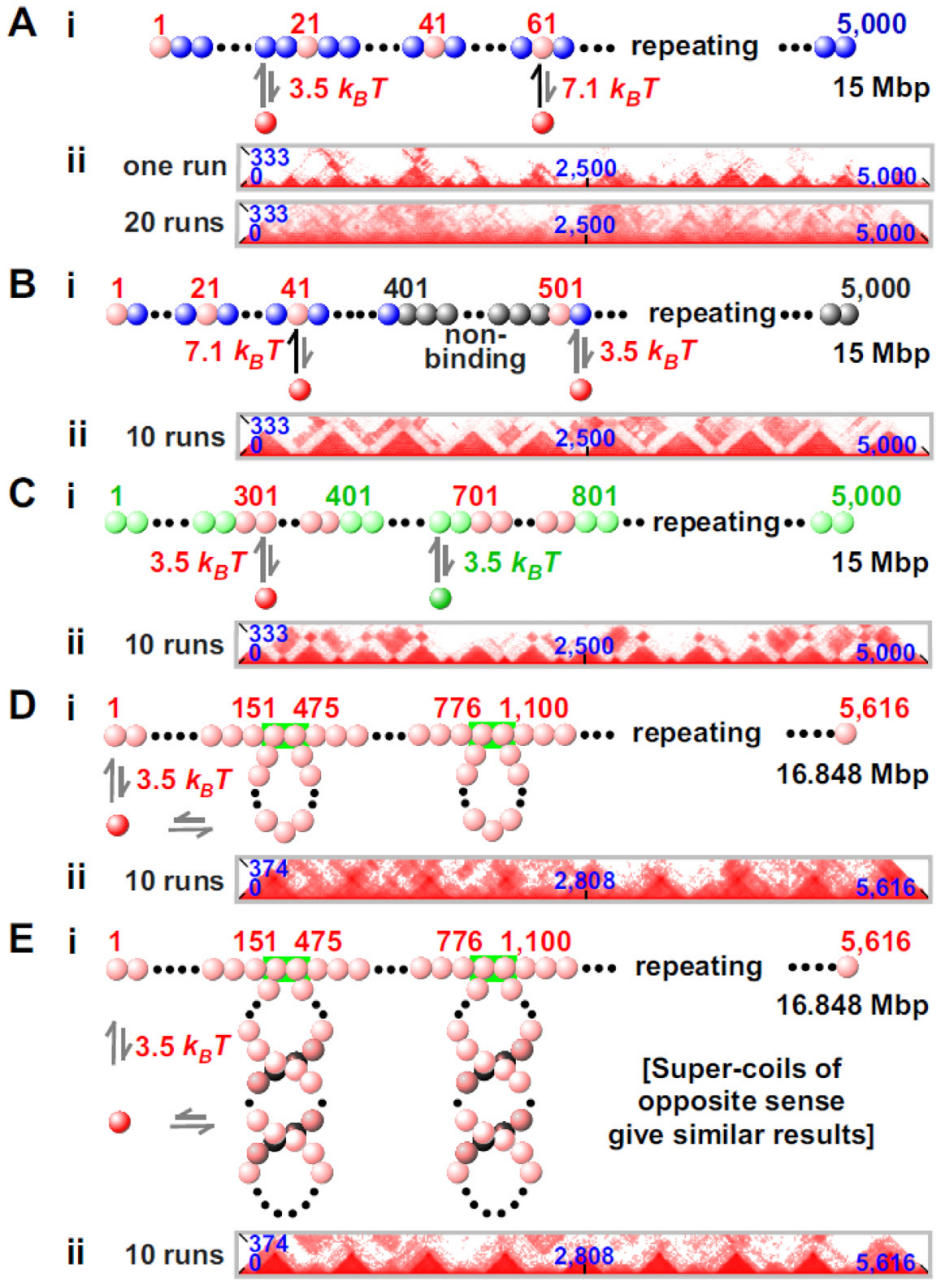
Domain formation. MD simulations were run as in Figure 1, unless stated otherwise (*n* = total number of runs). In contact maps, only regions around the (horizontally-placed) diagonal are shown; axes give bead numbers (blue). [Supplementary Figure S7 shows complete contact maps.] (**A**) Clustering of factors does not necessarily lead to domains. (i) Red factors bind with high-affinity to pink beads (one beads every 20), and with low affinity to blue beads. (ii) Although pyramids are seen in the contact map after 1 run, averaging data from 20 runs blurs patterns. (**B**) Gene deserts. (i) Blocks of 400 binding beads (blue and pink; one bead every 20 is pink) alternate with blocks of 100 non-binding beads (gray); red factors bind to blue and pink beads with low and high affinity, respectively. (ii) Each pyramid coincides with a block of pink and blue beads, and is separated from the next by a disordered region. (**C**) Hetero- and eu-chromatin. (i) Blocks of 300 light-green and 100 pink beads alternate; red and green factors bind to pink and light-green beads, respectively. (ii) Large pyramids alternate with small ones, reflecting reproducible assembly of blocks into domains. (**D**) Loops. (i) The fiber is pre-organized into loops by forcing selected beads (green rectangles) to bind irreversibly; this results in 324-bead loops separated by 300 unlooped beads (plus 150 unlooped ones at each end). All beads are pink and red factors can bind to any bead. Loops are initially torsionally relaxed (i.e. with linking number, Lk, equal to 0), we assume that the linking number is conserved in each loop throughout the simulation (for better comparison with (**E**) below). Nevertheless, we have checked that very similar results are obtained if this assumption is relaxed. (ii) Pyramids are less well defined than in (B and C), but nevertheless tend to coincide with loops (see also Supplementary Figure S9). (E) Supercoiled loops. (i) As (D), but each loop has a linking number of +32. (ii) Loops form pyramids that are more distinct than in (D).

**Figure 4.**
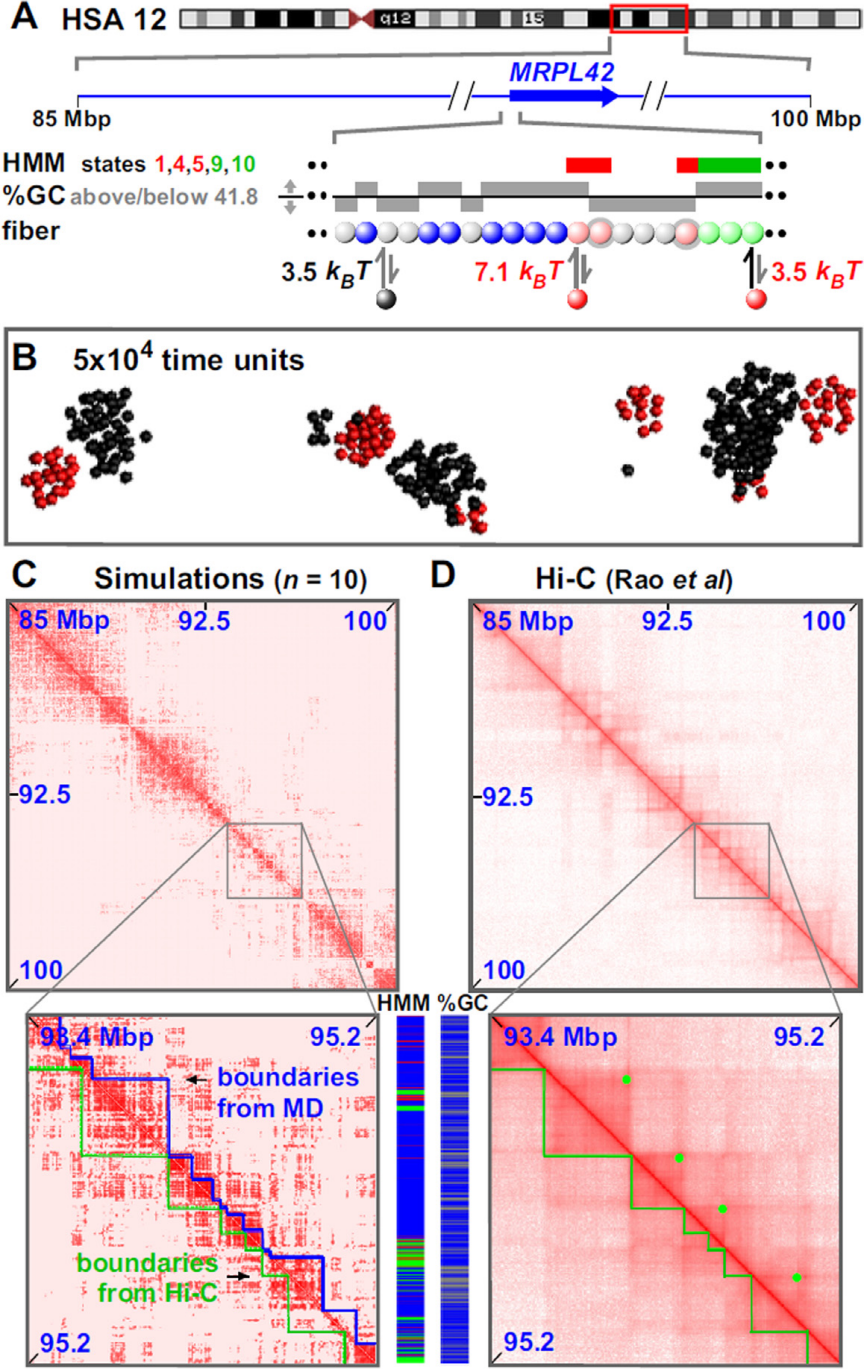
Simulating 15 Mbp of chromosome 12 in GM12878 cells. Conditions as Figure 1, with exceptions indicated (chromatin concentration now 0.01%). (**A**) Overview. The ideogram (red box gives region analyzed) and Broad ChromHMM track (colored regions reflect chromatin states) are from the UCSC browser; the zoom illustrates the MRPL42 promoter. Beads (1 kbp) are colored according to HMM state and GC content (blue—non-binding; pink—states 1 + 4 + 5, *n* = 600; light-green—states 9 + 10, *n* = 880; gray <41.8% GC, *n* = 10 646). Red factors (*n* = 300) bind to (active) pink and light-green beads with high and low affinities, respectively; black (heterochromatin-binding) proteins (*n* = 3000) bind to gray beads. In the zoom, two pink beads (gray halos) bind both red factors and black proteins. (**B**) Snapshot (without chromatin) of central region after 5 × 10^4^ time units; most clusters contain factors/proteins of one color. Long runs of gray beads form large black clusters. (**C** and **D**) Contact maps from simulations (7 kbp binning) and Hi-C (10 kbp binning; (7)). In zooms, blue and green lines mark boundaries determined by visual inspection of data from simulations or Hi-C, and dots in D mark loops found using the Janus plot (Supplementary Figure S9A). Tracks of HMM state and %GC (colored as in A) illustrate correlations with domains and boundaries.

**Figure 5.**
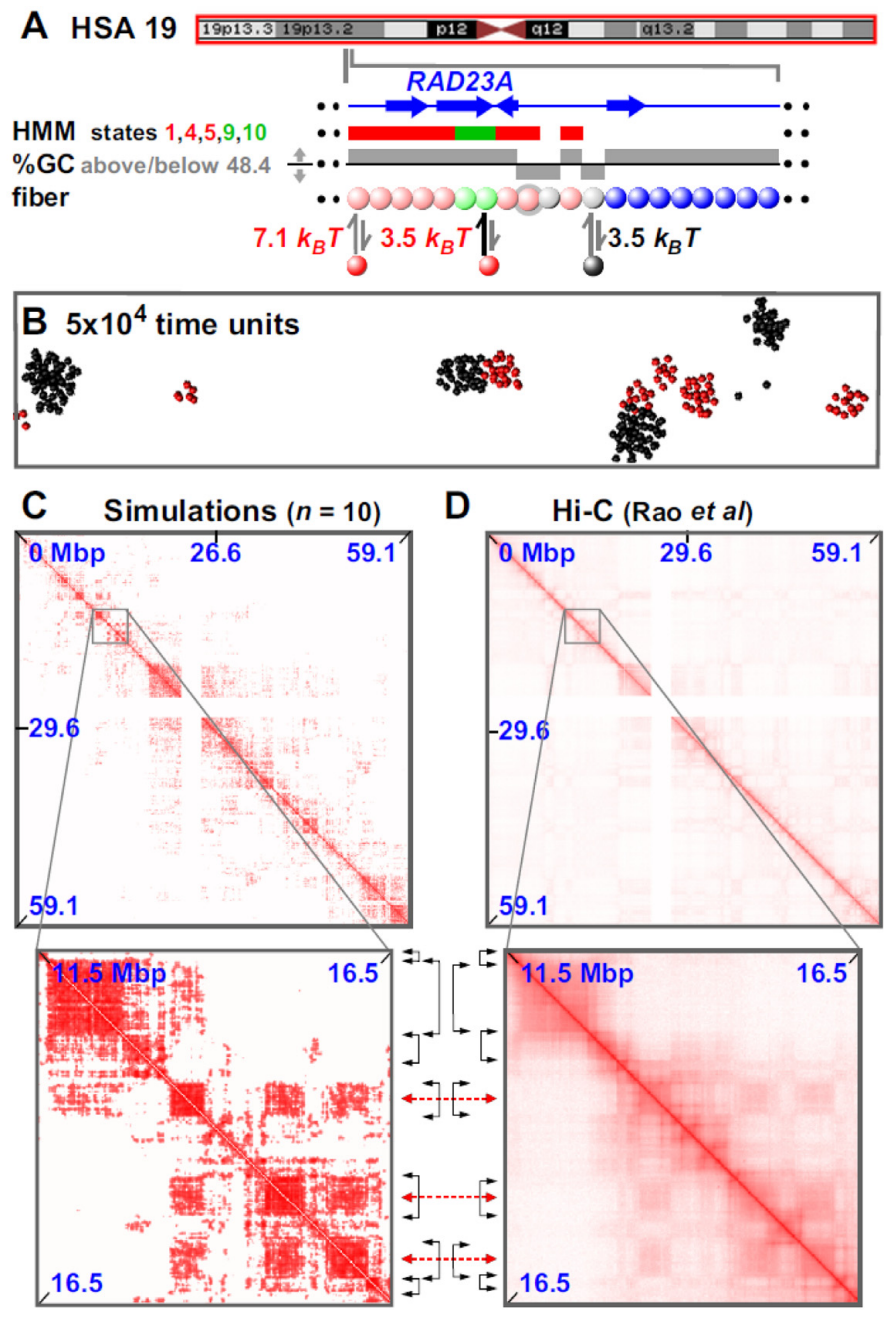
Simulating chromosome 19 in GM12878 cells. Conditions as Figure 4, with exceptions indicated. (**A**) Overview. The ideogram (red box indicates whole chromosome simulated) and HMM track (colored regions reflect chromatin states) are from the UCSC browser; the zoom illustrates the region around RAD23A. Beads (3 kbp) are colored according to HMM state and GC content (blue—non-binding; pink—states 1 + 4 + 5, *n* = 2473; light-green—states 9 + 10, *n* = 2686; gray <48.4% GC, *n* = 9472). Red factors (*n* = 400) bind to (active) pink and light-green beads with high and low affinities, respectively; black (heterochromatin-binding) proteins (*n* = 4000) bind to gray beads. In the zoom, two pink beads (gray halos) bind both red and black factors. (**B**) Snapshot (without chromatin) of central region after 5 × 10^4^ units; most clusters contain factors (or proteins) of one color. (**C** and **D**) Contact maps (21 and 20 kbp binning for data from simulations and Hi-C). Between zooms, black double-headed arrows mark boundaries of prominent domains (on the diagonal) and red double-headed ones the centers of off-diagonal blocks making many inter-domain contacts (boundaries and domains detected via the difference plot aided by visual inspection).

In our simulations, one bead corresponds either to 3 (Figures 1–3, 5) or 1 kbp (Figure 4 and Supplementary Figure S12). These correspond to a size of 30 and 20.8 nm respectively (assuming a 30-nm fiber has a packing density of 1 kbp/10 nm, and the two bead sizes contain the same volume density of DNA). Persistence length was 3 σ (representing a flexible polymer) and one time unit corresponds to 0.6 or 0.2 ms (for 3 or 1 kbp beads; calculated assuming a viscosity of 10 cP, or 10-fold larger than water).

## RESULTS

### Chromatin fibers interacting with bi- or multivalent factors spontaneously assemble into clusters

To begin, we study a ‘toy model’ where a ‘chromatin fiber’ of 15 Mbp is represented by 5000 30-nm beads diffusing amongst 30-nm ‘transcription factors’ (Figure 1A). Initially, ‘transcription factors’ (hereafter factors) have no affinity for any bead in the fiber (which follows a self-avoiding random walk), but then binding is ‘switched’ on so they now have a high affinity for some of the chromatin beads (pink) and a low affinity for all others. This choice emulates the tight binding of transcription factors to cognate sites and non-specific binding elsewhere. For simplicity, we first consider the case in which high-affinity beads are regularly spaced (every 20 beads in Figure 1A). While this toy model is too naive to represent real chromosomes (or even chromosomal fragments), we start with it because it allows us to introduce our modeling framework in a simple situation and to highlight some important general principles which are found in the more realistic models described later.

Importantly, in our simulations factors can bind to two (or more) beads, and affinities are just large enough to favor binding. Consequently, a factor often binds to a low-affinity site, dissociates and rebinds nearby. As this process repeats, the factor may reach a high-affinity site and remain bound long enough to stabilize a loop (Figure 1Ai); bound factors now spontaneously cluster (Figure 1Aii, 1Aiii; Supplementary Movies SM1 and 2). The force driving analogous clustering after non-specific binding was dubbed the ‘bridging-induced attraction’; it operates even though no explicit attraction between factors or between beads was specified, and it was not seen with monovalent factors or irreversible binding (32). Earlier work also shows such clustering occurs with chromatin fibers with 20-nm thickness (32), so the results we now present should also apply to fibers of this (or different) size.

We next examine some properties of the system. As binding compacts the fiber, and as beads in/around each cluster make many contacts, blocks of red pixels are seen along the diagonal in the resulting contact map (Figure 1Aiv)—similar to Hi-C data (2). After switching on binding, clusters form in <1 min (one simulation time unit is 0.6 ms, calculated assuming a nuclear viscosity of 10 cP) and the fraction of pink beads in clusters increases rapidly (Figure 1Av). [We define two beads to be in the same cluster if centers lie within 90 nm.] Small clusters then slowly grow to reach a steady-state size with 12 factors/cluster (Figure 1Av), when the entropic cost of gathering loops together (which scales non-linearly with loop number (48)) limits further growth. It is likely that such ‘coarsening’ is also dynamically hindered, as merging two clusters of loops (even when thermodynamically favored), would require passage over a free-energy barrier due to inter-loop interactions. Similar arrested coarsening is found in all cases described. While we cannot rule out that there may still be some very slow evolution or rearrangement in the system, we have verified that global indicators such as the gyration radius also reach a steady state (Supplementary Figure S1A).

It is also of interest to characterize in more detail the structure of the chromatin contact network associated with the clusters of factors (or of binding sites). In particular, ‘rosettes’ of loops are often found in models of chromosomes (49,50); therefore we developed a suitable plot—a ‘rosettogram’—to assess how many existed in our simulations (Supplementary Figure S1B). In a rosettogram, there is a row for every cluster, and a column for every high-affinity bead in a cluster (other beads are not shown); then, a red pixel marks the presence of a binding bead in a cluster, and increasing numbers of abutting red pixels in a row reflect increasing numbers of loops (‘petals’) involving near-neighbor high-affinity sites. In Figure 1Avi, the first cluster includes beads from both ends and two internal segments. However, the second organizes a ‘perfect’ rosette with 14 petals around high-affinity beads 21, 41, 61, …, 281; here, contacts display ‘transitivity’ (7), with one loop running from bead 21 to 41, another from 41 to 61 and a third from 21 to 61 via 41. In contrast, ‘overlapping loops’ (of the type running directly from bead 21 to 61 and from 41 to 81) are rarely seen here or in Hi-C data (7). As most rows contain adjacent pixels, and as 80% pink beads share a cluster with a nearest-neighbor pink bead, a measure of the disorganization (i.e. the disorganized fraction, *f*_*d*_) is low (Supplementary Figure S1B; Supplementary Table S1). In other words, rosettes and local contacts are relatively common in this toy model.

We now examine how randomly scattering the same number of high-affinity sites along a fiber affects contacts. The result is a more regular string of rosettes (Figure 1B; Supplementary Figure S2B gives further details) with a lower *f_d_* value, presumably because gaps between successive binding sites are exponentially distributed so that binding sites are naturally clustered nearer together in 1D genomic space (this is the so-called ‘Poisson clumping’). If low-affinity sites are omitted (Supplementary Figure S2A), the contact map, rosettogram and *f*_*d*_ all point to a more disorganized structure, which is less similar to a regular string of rosettes with respect to those of Figure 1A or B. In both cases, clusters still form. [This is also the case with a higher concentration of chromatin (i.e. in the ‘semi-dilute’ regime (51), see below and Supplementary Figure S5).]

### Different transcription factors form into different clusters

Binding of different factors to different beads is now analyzed, again starting for simplicity from a toy model to highlight general principles. In Figure 2Ai, green factors interact only with light-green beads and red ones only with pink beads. Again, no attraction is specified between factors, or between beads. Remarkably, clusters now contain only red factors—or only green ones—but rarely both (mixed clusters are not seen at the end of this simulation; Figure 2Aii, Supplementary Movies SM3 and 4). As before, clusters reach a steady state, but now with only ∼8.1 bound factors/cluster; the regular alternation of green and red binding sites renders cluster merging entropically more costly. Moreover, the contact map, rosettogram and *f*_*d*_ all point to a more disorganized structure than those seen previously; for example, there are now many ‘overlapping’ loops where the fiber passes back and forth between a cluster of red factors to another with green ones (Figure 2Aiii and iv). As expected, mixed clusters result if factors share binding sites (Supplementary Figure S3).

Such self-organization into structures rich in certain DNA-binding proteins but not others is commonplace in nuclear biology (see ‘Discussion’ section). But what might drive this self-assembly into ‘specialized’ clusters in the absence of any explicit interaction between factors, or between beads? We suggest there are both entropic and kinetic drivers (32), and that the following one is important. Thus, early during the simulation in Figure 2A, a structure like that in Figure 2B might arise. Red protein 1 is tightly bound to two pink beads, and so will rarely dissociate from the cluster; however, if it does it is likely to bind to a nearby pink bead (as there are so many). Further, as red protein 2 and binding bead *b* diffuse by, both are likely to join the same cluster (again because of the high local concentration of appropriate binding sites and factors). We are now in a positive feedback loop: the local concentration of red factors and pink beads makes it unlikely either will escape and likely that more of both will be caught as they diffuse by. For the same reason, green protein 3 is likely to bind to the right-hand cluster and this cluster will tend to grow as other green factors and light-green sites are caught. Red and green clusters will inevitably be separate in 3D space because their cognate binding sites are separate in 1D sequence space, and cluster growth is limited when the entropic costs of bringing together ever-more loops becomes prohibitive (similarly to the case of binding sites with a single color analyzed in Figure 1).

In Figure 2A, red and green binding beads alternate, and overlapping loops pass back and forth between red and green clusters. Rosettes with many transitive loops instead result if 200-bead blocks containing 10 light-green beads (spaced every 20 beads) alternate with similar blocks containing pink beads (Supplementary Figure S4). As there are fewer binding beads of one color per block than the ∼12 often found in a cluster in the analogous simulation in Figure 1A, successive blocks can form successive red and green clusters. Unsurprisingly, the 1D organization determines rosette and loop type.

Distinct clusters also form if more factors and more fibers are introduced. In Figure 2C and Supplementary Figure S5, five differently-colored factors bind to distinct cognate sites scattered randomly along 20 fibers. Distinct clusters again form; 53% contain factors of only one color, and in >80% more than 80% binding beads have the same color (Supplementary Figure S5v). Such clustering could underlie the high number of contacts seen between co-regulated genes that utilize the same factors (10,15–18). Inter-fiber contacts are rare (Supplementary Figure S5iv), as in Hi-C data (2). However, they constitute a higher fraction if just contacts made by binding beads in clusters are considered (Supplementary Figure S5iv). This is reminiscent of contacts made by active genomic regions; for example, most contacts made by (active) *SAMD4A* are inter-chromosomal (assessed by 4C (18)), as are most (active) sites binding estrogen receptor α (15). [Supplementary Figure S5v gives effects of the threshold used to define contacts on contact frequencies.]

The affinities of transcription factors for cognate sites are often tightly regulated, frequently by post-translational modification. Changing factor affinity was simulated using a fiber in which one bead every 20 was yellow, with regular spacing (Supplementary Figure S6). Initially, red factors bind to yellow beads and red clusters form. Then, we switch on an attraction between green factors and yellow beads that is stronger than the red-yellow attraction; consequently, green factors compete effectively with red ones, and red/green and all-green clusters develop. This provides a precedent for how one nuclear body (e.g. a transcription factory) might evolve into another.

### Forming domains

Topological domains are recognized as ‘pyramids’ in contact maps prepared using data from Hi-C (3–4,8)) or simulations (30,33–34,41–42). Our simulations demonstrate that the pattern of binding beads in our fibers determines whether pyramids are seen. For example, Figure 3A illustrates a partial contact map obtained using data from Figure 1A. As clusters appear stochastically and tend to persist, a specified bead often clusters with different partners in different simulations. Then, pyramidal patterns, which are visible in a single run (analogous to Hi-C data from a single cell), become blurred on averaging results from progressively more simulations (Figure 3Aii). [Supplementary Figure S7 gives complete contact maps for all simulations in Figure 3.]

In a homogeneous fiber, pyramids disappear on averaging because domains form stochastically; however, if the fiber is suitably patterned, domain boundaries form at specific locations. Barbieri *et al*. (30) have previously shown that associating a different factor with each domain can drive their formation; here we investigate whether other mechanisms, which require fewer types of factors, can also lead to domains. For example, if long binding blocks in which one bead every 20 is pink (binding red factors strongly) are interleaved with short blocks containing non-binding gray beads (representing gene-poor ‘deserts’), pink and blue beads cluster but gray ones do not; then, many contacts are seen between pink and blue beads to give pyramids sitting exactly on long segments (Figure 3B). Here, boundaries between domains are located within gray segments. Domains are also seen if segments containing 300 successive pink beads (binding red factors) are interleaved with shorter segments containing 100 light-green beads (binding green factors); in this case, a repeating pattern of large and small pyramids is seen, with boundaries between blocks of differently-colored beads (Figure 3C). This simulation could mimic binding of polymerizing complexes and HP1α (hetero-chromatin protein 1α) to repeats of eu- and hetero-chromatin (52). [These results confirm and extend those obtained using Monte-Carlo simulations of just one segment of each type (30,38).] These runs of binding beads give larger clusters (i.e. ∼40 red factors/cluster, and ∼15 green factors/cluster). When the fiber is forced permanently into loops (perhaps maintained by CTCF)—and if red factors can bind to any bead—pyramids (which are more blurred than in Figure 3B and C) tend to sit over each loop (Figure 3D (21)); this is reminiscent of Hi-C data (3,7,21). If loops are preformed into left-handed (or right-handed) inter-wound supercoils (53), pyramids are more sharply defined (Figure 3E; these results are also consistent with those obtained in (21,33)). Therefore, domains can form in a non-uniform genomic landscape (Figure 3B and C), and if there are loops (Figure 3D and E).

Because our fibers form many (>10) domains, we can analyze contact maps away from the diagonal: these clearly show that in all cases where domains form, inter-domain interactions are weaker than intra-domain ones (compare Supplementary Figure S7B–E)—as in Hi-C data. [Note that many domain:domain interactions seen after one run (Supplementary Figure S7F) disappear (or become fainter) after averaging data from many runs (Supplementary Figure S7B).] The results obtained with these simple models suggest that there are multiple ways of creating domains. Thus, models in Figure 3B, C and E (and, to a lesser degree, in Figure 3D), all lead to the formation of domains; the models in Figures 3D and E would also yield ‘loop domains’ (as described in (7)), because contacts at the base of the loop are seen with an increased probability. The domains in the model in Figure 3B are separated by non-binding regions, which is qualitatively similar to what is observed in simulations of the *C. crescentus* chromosome (21); the model in Figure 3C is instead consistent with the observations that many Hi-C boundaries coincide with epigenetic boundaries between active and inactive regions (5). Therefore, each model in Figure 3B-E illustrates a possible mechanism underlying the formation of topological domains. Presumably, all will be active in practice. For simplicity, in the simulations of human chromosomes which follow, pre-formed loops and/or supercoiling are not included.

### Some characteristics of domains

The probability that two loci yield a Hi-C contact decreases as the number of intervening base pairs increases (2) and the exponent (α) in the power law varies from −0.5 (in HeLa (6)) to −1.6 (in embryonic stem cells (30)). [α = −1 in the fractal globule model (2,6).] In all simulations in Figure 3 (except for Figure 3A), there are two regimes—below and above the domain size (the largest of the two domains appears to set the scale) with α between −0.6 and −1 (strong interactions within a pyramid/domain) or close to −2 (weaker interactions between pyramids/domains; Supplementary Figure S8). Therefore our values are similar to those seen experimentally, which also show different apparent exponents for intra- and inter-domain interactions (54). Our results are further consistent with the exponent (seen in simulations of uniform fibers) varying with protein number and affinity (30).

We next describe various approaches to identify domain boundaries (Supplementary Figure S9). Many current approaches are based on what we will call a Janus plot (Supplementary Figure S9), which in its simplest form quantifies all contacts that one bead makes with others to the right or the left in 1D genomic space. In Figure 3D, peaks in the two resulting plots correlate well with the left and right tethers of a loop (Supplementary Figure S9A). By subtracting signal from the two plots, we obtain a ‘difference plot’ (i.e. the number of contacts to the right minus the number of contacts to the left). At a boundary, we expect a bead to switch its contacts, from mostly leftward to mostly rightward; consequently, boundaries are found at points where signal in the difference plot crosses zero with an upward derivative (Supplementary Figure S9B). This is essentially the method used in (3). In the case of Figure 3D, this approach finds domains within loops, and boundaries somewhere in the linear region between them. A more accurate determination is possible by locating the peaks of the derivative of the difference plot (the ‘insulator’ signal in Supplementary Figure S9C). This boundary-finding algorithm is elegant and works well with highly-sampled contact maps (as in Figure 3). However, it works less well with sparser data from simulations in the next Section and Hi-C data, where the ‘difference’ plot gives better results (so we use it in what follows, aided by visual inspection to fine-tune boundary positions).

### Modeling selected regions of the human genome

Finally, we examine whether our simulations can reproduce experimental interaction maps. We have shown above that specific 1D patterning of binding sites drives 3D structures, and that multiple factors lead to the formation of segregated clusters. We therefore investigate whether binding of just two proteins to ‘active’ and ‘inactive’ beads on a 15-Mbp fiber (representing part of chromosome 12 in GM12878) could fold the genome appropriately (Figure 4A; Supplementary Movies SM5 and 6). Active regions were selected using the Broad ChromHMM track on the UCSC (University of California at Santa Cruz) browser (47), and beads (1 kbp) representing active promoters or strong enhancers (states 1, 4, 5)—and the bodies of active transcription units (states 9, 10)—were colored pink and light-green, respectively. These pink and light-green beads bind red factors (transcription factors, polymerizing complexes) with high and low affinities. Inactive heterochromatin was represented by gray beads that bind black proteins (e.g. dimers of HP1α (52)). Heterochromatic beads were identified as those having a low GC content—an excellent and flexible marker (in principle, choice of threshold can allow any fraction of the region of interest to be classified as heterochromatin). Here, <41.8% GC was chosen as the threshold, as this led to the same percentage of heterochromatin in the 15 Mbp as that marked by state 13 (generic heterochromatin) in the HMM track. Other beads (blue) were non-binding. [We have found that for a wide range of choices for the % GC threshold, this has only has a minor effect on the contact maps resulting from the simulations (Supplementary Figure S10).] As before, distinct clusters of bound red or black proteins develop with ∼14 or ∼190 proteins/cluster, respectively (long runs of adjacent gray beads form the larger clusters; Figure 4B). The resulting contact map was strikingly similar to the Hi-C one (7), with simulations correctly predicting 75% of the Hi-C domain boundaries to within 100 kbp (Figure 4C and D; Supplementary Figure S11A, B and C; Supplementary Table S2). Boundaries were in this case found using the difference plot aided by visual inspection (Supplementary Figure S11); purely automated detection correctly locates ∼59% Hi-C boundaries within 100 kbp, which is still statistically significant (this is an underestimate due to algorithmic errors, some examples of which are noted in Supplementary Figure S11). Simulations also yield a more-ordered rosettogram than any seen previously (Supplementary Figure S11D); this is consistent with evolution selecting for a genetic and epigenetic sequence that favors ordered rosettes. Similar results were obtained with a 15-Mbp segment of a different chromosome (chr6) in a different cell type (Supplementary Figure S12). [Here, heterochromatic regions were defined using either %GC or HMM state 13, but fewer boundaries were correctly reproduced using the latter (Supplementary Figure S12E).]

These successes prompted us to model a whole 59-Mbp chromosome (chr19; see Figure 5 and Supplementary Movie SM7). Active and inactive beads (each now representing 3 kbp) were defined as before, and heterochromatic ones using <48.4% GC (to reproduce the fraction of the chromosome bearing the state 13 mark). Now, 85% domain boundaries are correctly reproduced to within 100 kbp (Figure 5 and Supplementary Figure S13A and B). Moreover, simulation boundaries are rich in ‘active’ and non-binding regions and depleted of ‘inactive’ ones (Supplementary Figure S13C). As before, the rosettogram and *f*_*d*_ point to a highly-ordered structure with many local contacts (Supplementary Figure S13D). At a higher level in the organization, 3D positioning of some domains next to others—reflected by off-diagonal blocks in contact maps—is sometimes reproduced in simulations (zooms in Figure 5C and D and Supplementary Figure S13A). Our simulations of the whole chromosome further indicate that small active domains seem to be often located at the periphery of larger inactive ones; they also suggest that active domains are more dynamic and mobile than inactive ones (see Supplementary Movie SM7).

## DISCUSSION

These MD simulations illustrate some emergent properties of a minimalist system that involves bivalent or multivalent ‘transcription factors’ (or ‘proteins’) binding specifically and transiently to cognate sites in a fiber (representing ‘chromatin’). First, bound factors spontaneously cluster—to compact the fibers (Figures 1 and 2). This self-organization occurs in the absence of any explicit interaction between factors or between beads, and it is driven by a combination of forces dubbed the ‘bridging-induced attraction’. Second, and more surprisingly, factors binding to distinct sites on the fiber self-assemble into distinct (segregated) clusters. For example, bound red and green factors self-assemble into clusters that contain only red factors, or only green ones—but rarely both (Figure 2A). These clusters arise because protein binding will inevitably yield clusters in different places in 3D space if—and only if—their cognate binding sites are spatially separated in 1D sequence space (Figure 2B). Our third and main result is that clustering organizes the loops caused by binding into topological domains (Figures 3–5). For example, binding of just two ‘proteins’ (transcription and HP1α complexes) to active and inactive regions in a 59-Mbp human chromosome folds the fiber to yield a contact map in which ∼85% of the Hi-C boundaries are both correctly placed and rich in the appropriate sequences (Figure 5, Supplementary Figure S13 and Supplementary Movie SM7). In other words, complexes bind locally to create loops, bound complexes cluster together with similar ones into rosettes, this folds the fiber globally into appropriate domains and domains pack against each other—all in the expected ways. Remarkably, then, this minimalist system generates structures that possess all the key features of interphase chromosomes outlined in the Introduction. Moreover, the clusters formed are reminiscent of nuclear structures like Cajal and promyelocytic leukemia bodies, which are each rich in distinct proteins that bind to different cognate DNA sequences (55); they also closely resemble nucleoplasmic transcription factories that each contain ∼10 active polymerizing complexes (56–58).

The binding energy of any one factor is small (roughly comparable to that in a few H-bonds), but extended genomic regions fold simply because so many are involved. Cluster formation and protein-driven chromosome organization also occurs quickly (within minutes according to our simulations, Figure 1Av). Once clusters form, they usually persist (Figure 1Av). However, the system can evolve when new factors appear (Supplementary Figure S6), much as a transcription factory can develop into a replication factory at the beginning of S phase (59), or into one specializing in transcribing responsive genes during the inflammatory response (when tumor necrosis factor α induces nuclear influx of nuclear factor κB (18)).

Contacts made as a results of such clustering involve sites both near and far apart on the fiber. Most contacts are local, and we observe two different types of structure. In active regions, high-affinity binding beads (at promoters and enhancers) tend to be scattered amongst low-affinity (active) beads, and this drives folding into rosettes with mainly local loops (49,50) (Supplementary Figures S11D and S13D). We suggest that rosettes (with many transitive loops) are favored over more disordered non-local structures (with many overlapping loops) partly because the entropic cost is less (60); rosettes are also likely to be kinetically favored when starting from knot-free structures (both in simulations and on exit from mitosis). In inactive regions, binding beads tend to be in long runs and this favors folding into compact, globular, structures (Figures 4 and 5).

In summary, the bridging-induced attraction provides a robust, simple and generic mechanism that can concentrate specific proteins bound to cognate sites into clusters, and fold interphase fibers in ways found *in vivo*. Then, the system must either spend energy to prevent the resulting clustering, or—as seems likely—it goes with the flow and uses other more or less familiar forces (charge interactions, H bonds, van der Waals, hydrophobic forces and the depletion attraction (22)) to organize those clusters. If so, we suggest that the particular folding pattern found in any one nucleus will be largely determined by which transcription factors bind to cognate sites and which bound factors then happen to co-cluster. We also expect that adding more proteins and fibers to our simple model will improve the concordance between contact maps obtained from simulations and Hi-C.

After the present work was completed, two studies proposed another model for forming looped domains through CTCF bridges (54,61). Both involve some loop-extruding mechanism driving domain formation, and they are appealing because they can account for the observation that CTCF bridging depends on the orientation of the cognate binding sites (7,62). However, this model requires some as yet undiscovered motor protein with a processivity sufficient to generate loops of hundreds of kbps. On the other hand, as discussed in Ref. (54), these studies do not address what might underlie the observed compartmentalization into active and inactive domains—which is naturally explained within our framework by binding of different factors to eu- and hetero-chromatin. Furthermore, knock-outs of CTCF have only minor effects on domain organization (63,64), which again suggests that this factor cannot be the sole organizer. The results of these knock-outs are also naturally explained by our model, as the compartmentalization is driven by bi- or multivalent factors (in addition to CTCF). Our work and those of Refs. (54,61) are therefore complementary, and it would be of interest to couple the two approaches together in the future.

## Supplementary Material

SUPPLEMENTARY DATA

## References

[1] Cavalli G., Misteli T. (2013). Functional implications of genome topology. Nat. Struct. Mol. Biol..

[2] Lieberman-Aiden E., van Berkum N.L., Williams L., Imakaev M., Ragoczy T., Telling A., Amit I., Lajoie B.R., Sabo P.J., Dorschner M.O. (2009). Comprehensive mapping of long-range interactions reveals folding principles of the human genome. Science.

[3] Dixon J.R., Selvaraj S., Yue F., Kim A., Li Y., Shen Y., Hu M., Liu J.S., Ren B. (2012). Topological domains in mammalian genomes identified by analysis of chromatin interactions. Nature.

[4] Nora E.P., Lajoie B.R., Schulz E.G., Giorgetti L., Okamoto I., Servant N., Piolot T., van Berkum N.L., Meisig J., Sedat J. (2012). Spatial partitioning of the regulatory landscape of the X-inactivation centre. Nature.

[5] Sexton T., Yaffe E., Kenigsberg E., Bantignies F., Leblanc B., Hoichman M., Parrinello H., Tanay A., Cavalli G. (2012). Three-dimensional folding and functional organization principles of the Drosophila genome. Cell.

[6] Naumova N., Imakaev M., Fudenberg G., Zhan Y., Lajoie B.R., Mirny L.A., Dekker J. (2013). Organization of the mitotic chromosome. Science.

[7] Rao S.S.P., Huntley M.H., Durand N.C., Stamenova E.K., Bochkov I.D., Robinson J.T., Sanborn A.L., Machol I., Omer A.D., Lander E.S. (2014). A 3D map of the human genome at kilobase resolution reveals principles of chromatin looping. Cell.

[8] Sexton T., Cavalli G. (2015). The role of chromosome domains in shaping the functional genome. Cell.

[9] Simonis M., Klous P., Splinter E., Moshkin Y., Willemsen R., de Wit E., van Steensel B., de Laat W. (2006). Nuclear organization of active and inactive chromatin domains uncovered by chromosome conformation capture-on-chip (4C). Nat. Genet..

[10] Li G., Ruan X., Auerbach R.K., Sandhu K.S., Zheng M., Wang P., Poh H.M., Goh Y., Lim J., Zhang J. (2012). Extensive promoter-centered chromatin interactions provide a topological basis for transcription regulation. Cell.

[11] Jin F., Li Y., Dixon J.R., Selvaraj S., Ye Z., Lee A.Y., Yen C.A., Schmitt A.D., Espinoza C.A., Ren B. (2013). A high-resolution map of the three-dimensional chromatin interactome in human cells. Nature.

[12] Zhang Y., Wong C.H., Birnbaum R.Y., Li G.L., Favaro R., Ngan C.Y., Lim J., Tai E., Poh H.M., Wong E. (2013). Chromatin connectivity maps reveal dynamic promoter-enhancer long-range associations. Nature.

[13] Heidari N., Phanstiel D.H., He C., Grubert F., Jahanbani F., Kasowski M., Zhang M.Q., Snyder M.P. (2014). Genome-wide map of regulatory interactions in the human genome. Genome Res..

[14] Mifsud B., Tavares-Cadete F., Young A.N., Sugar R., Schoenfelder S., Ferreira L., Wingett S.W., Andrews S., Grey W., Ewels P.A. (2015). Mapping long-range promoter contacts in human cells with high-resolution capture Hi-C. Nat. Gen..

[15] Fullwood M.J., Liu M.H., Pan Y.F., Liu J., Xu H., Bin Mohamed Y. (2009). An oestrogen-receptor-alpha-bound human chromatin interactome. Nature.

[16] Schoenfelder S., Sexton T., Chakalova L., Cope N.F., Horton A., Andrews S., Kurukuti S., Mitchell J.A., Umlauf D., Dimitrova D.S. (2010). Preferential associations between co-regulated genes reveal a transcriptional interactome in erythroid cells. Nat. Genet..

[17] Yaffe E., Tanay A. (2011). Probabilistic modeling of Hi-C contact maps eliminates systematic biases to characterize global chromosomal architecture. Nat. Genet..

[18] Papantonis A., Kohro T., Baboo S., Larkin J., Deng B., Short P., Tsutsumi T., Taylor S., Kanki Y., Kobayashi M. (2012). TNFα signals through specialized factories where responsive coding and micro-RNA genes are transcribed. EMBO J..

[19] Nagano T., Lubling Y., Stevens T.J., Schoenfelder S., Yaffe E., Dean W., Laue E.D., Tanay A., Fraser P. (2013). Single-cell Hi-C reveals cell-to-cell variability in chromosome structure. Nature.

[20] Hsieh T.-H.S., Weiner A., Lajoie B., Dekker J., Friedman N., Rando O.J. (2015). Mapping nucleosome resolution chromosome folding in yeast by micro-C. Cell.

[21] Le T.B., Imakaev M.V., Mirny L.A., Laub M.T. (2013). High-resolution mapping of the spatial organization of a bacterial chromosome. Science.

[22] Marenduzzo D., Micheletti C., Cook P.R. (2006). Entropy-driven genome organization. Biophys. J..

[23] Rosa A., Everaers R. (2008). Structure and dynamics of interphase chromosomes. PLoS Comput. Biol..

[24] Nicodemi M., Panning B., Prisco A. (2008). The colocalization transition of homologous chromosomes at meiosis. Phys. Rev. E.

[25] Duan Z., Andronescu M., Schutz K., McIlwain S., Kim Y.J., Lee C., Shendure J., Fields S., Blau C.A., Noble W.S. (2010). A three-dimensional model of the yeast genome. Nature.

[26] Junier I., Martin O., Képès F. (2010). Spatial and topological organization of DNA chains induced by gene co-localization. PLoS Comput. Biol..

[27] de Vries R. (2011). Influence of mobile DNA-protein-DNA bridges on DNA configurations: coarse-grained Monte-Carlo simulations. J. Chem. Phys..

[28] Kalhor R., Tjong H., Jayathilaka N., Alber F., Chen L. (2011). Genome architectures revealed by tethered chromosome conformation capture and population-based modeling. Nat. Biotechnol..

[29] Rousseau M., Fraser J., Ferraiuolo M.A., Dostie J., Blanchette M. (2011). Three-dimensional modeling of chromatin structure from interaction frequency data using Markov chain Monte Carlo sampling. BMC Bioinformatics.

[30] Barbieri M., Chotalia M., Fraser J., Lavitas L.M., Dostie J., Pombo A., Nicodemi M. (2012). Complexity of chromatin folding is captured by the strings and binders switch model. Proc. Natl. Acad. Sci. U.S.A..

[31] Baù D., Marti-Renom M.A. (2012). Genome structure determination via 3C-based data integration by the Integrative Modeling Platform. Methods.

[32] Brackley C.A., Taylor S., Papantonis A., Cook P.R., Marenduzzo D. (2013). Nonspecific bridging-induced attraction drives clustering of DNA-binding proteins and genome organization. Proc. Natl. Acad. Sci. U.S.A..

[33] Benedetti F., Dorier J., Burnier Y., Stasiak A. (2014). Models that include supercoiling of topological domains reproduce several known features of interphase chromosomes. Nucleic Acids Res..

[34] Jost D., Carrivain P., Cavalli G., Vaillant C. (2014). Modeling epigenome folding: formation and dynamics of topologically associated chromatin domains. Nucleic Acids Res..

[35] Tark-Dame M., Jerabek H., Manders E.M.M., Heermann D.W., van Driel R. (2014). Depletion of the chromatin looping proteins CTCF and cohesin causes chromatin compaction: insight into chromatin folding by polymer modelling. PLoS Comput. Biol..

[36] Trieu T., Cheng J. (2014). Large-scale reconstruction of 3D structures of human chromosomes from chromosomal contact data. Nucleic Acids Res..

[37] Cheng T.M.K., Heeger S., Chaleil R.A.G., Matthews N., Stewart A., Wright J., Lim C., Bates P.A., Uhlmann F. (2015). A simple biophysical model emulates budding yeast chromosome condensation. Elife.

[38] Hofmann A., Heermann D.W. (2015). The role of loops on the order of eukaryotes and prokaryotes. FEBS Lett..

[39] Johnson J., Brackley C.A., Cook P.R., Marenduzzo D. (2015). A simple model for DNA bridging proteins and bacterial or human genomes: bridging-induced attraction and genome compaction. J. Phys. Condens. Matter.

[40] Junier I., Spill Y.G., Marti-Renom M.A., Beato M., le Dily F. (2015). On the demultiplexing of chromosome capture conformation data. FEBS Lett..

[41] Trussart M., Serra F., Baù D., Junier I., Serrano L., Marti-Renom M.A. (2015). Assessing the limits of restraint-based 3D modeling of genomes and genomic domains. Nucleic Acids Res..

[42] Zhang B., Wolynes P.G. (2015). Topology, structures, and energy landscapes of human chromosomes. Proc. Natl. Acad. Sci. U.S.A..

[43] Giorgetti L., Galupa R., Nora E.P., Piolot T., Lam F., Dekker J., Tiana G., Heard E. (2014). Predictive polymer modeling reveals coupled fluctuations in chromosome conformation and transcription. Cell.

[44] Dekker J., Marti-Renom M.A., Mirny L.A. (2013). Exploring the three-dimensional organization of genomes: interpreting chromatin interaction data. Nat. Rev. Genet..

[45] Serra F., Di Stefano M., Spill Y.G., Cuartero Y., Goodstadt M., Baù D., Marti-Renom M.A. (2015). Restraint-based three-dimensional modeling of genomes and genomic domains. FEBS Lett..

[46] Plimpton S. (1995). Fast parallel algorithms for short-range molecular dynamics. J. Comp. Phys..

[47] Ernst J., Kheradpour P., Mikkelsen T.S., Shoresh N., Ward L.D., Epstein C.B., Zhang X., Wang L., Issner R., Coyne M. (2011). Mapping and analysis of chromatin state dynamics in nine human cell types. Nature.

[48] Duplantier B. (1989). Statistical mechanics of polymer networks of any topology. J. Stat. Phys..

[49] Pienta K.J., Coffey D.S. (1984). A structural analysis of the role of the nuclear matrix and DNA loops in the organization of the nucleus and chromosome. J. Cell Sci. Suppl..

[50] Cook P.R. (1995). A chromomeric model for nuclear and chromosome structure. J. Cell Sci..

[51] Doi M., Edwards S.F. (1986). The Theory of Polymer Dynamics.

[52] Kilic S., Bachmann A.L., Bryan L.C., Fierz B. (2015). Multivalency governs HP1α association dynamics with the silent chromatin state. Nat. Commun..

[53] Gilbert N., Allan J. (2014). Supercoiling in DNA and chromatin. Curr. Opin. Genet. Dev..

[54] Sanborn A.L., Rao S.S.P., Huang S.C., Durand N.C., Huntley M.H., Jewett A.I., Bochlov I.D., Chinappan D., Cutkosky A., Li J. (2015). Chromatin extrusion explains key features of loop and domain formation in wild-type and engineered genomes. Proc. Natl. Acad. Sci. U.S.A..

[55] Sleeman J.E., Trinkle-Mulcahy L. (2014). Nuclear bodies: new insights into assembly/dynamics and disease relevance. Curr. Opin. Cell Biol..

[56] Pombo A., Jackson D.A., Hollinshead M., Wang Z., Roeder R.G., Cook P.R. (1999). Regional specialization in human nuclei: visualization of discrete sites of transcription by RNA polymerase III. EMBO J..

[57] Cook P.R. (1999). The organization of replication and transcription. Science.

[58] Papantonis A., Cook P.R. (2013). Transcription factories; genome organization and gene regulation. Chem. Rev..

[59] Hassan A.B., Errington R.J., White N.S., Jackson D.A., Cook. P.R. (1994). Replication and transcription sites are colocalized in human cells. J. Cell Sci..

[60] Marenduzzo D., Orlandini E. (2009). Topological and entropic repulsion in biopolymers. J. Stat. Mech..

[61] Fudenberg G., Imakaev M., Lu C., Goloborodko A., Abdennur N., Mirny L.A. (2015). Formation of chromosomal domains by loop extrusion.

[62] Guo Y., Xu Q., Canzio D., Shou J., Li J.H., Gorkin D.U., Jung I., Wu H.Y., Zhai Y.N., Tang Y.X. (2015). CRISPR inversion of CTCF sites alters genome topology and enhancer/promoter function. Cell..

[63] Zuin J., Dixon J.R., can der Reijden M.I.J.A., Ye Z., Kolovos P., Brouwer R.W.W., van de Corput M.P.C., van de Werken H.J.G., Knoch T.A., van IJcken W.F.J. (2014). Cohesin and CTCF differentially affect chromatin architecture and gene expression in human cells. Proc. Natl. Acad. Sci. U.S.A..

[64] Hou C., Dale R., Dean A. (2010). Cell type specificity of chromatin organization mediated by CTCF and cohesin. Proc. Natl. Acad. Sci. U.S.A..

