# Supplementary material for: Simulated binding of transcription factors to active and inactive regions folds human chromosomes into loops, rosettes and topological domains
Source: Nucleic Acids Res. 2016 Apr 8;44(8):3503–12. doi: 10.1093/nar/gkw135 (PMC4856988; doi:10.1093/nar/gkw135)
Supplement: SUPPLEMENTARY DATA [file supp_44_8_3503__index.html]

Simulated binding of transcription factors to active and inactive regions folds human chromosomes into loops, rosettes and topological domains — Simulated binding of transcription factors to active and inactive regions folds human chromosomes into loops, rosettes and topological domains — SUPPLEMENTARY DATA 

# Simulated binding of transcription factors to active and inactive regions folds human chromosomes into loops, rosettes and topological domains

## SUPPLEMENTARY DATA

- SUPPLEMENTARY DATA
- SUPPLEMENTARY DATA
- SUPPLEMENTARY DATA
- SUPPLEMENTARY DATA
- SUPPLEMENTARY DATA
- SUPPLEMENTARY DATA
- SUPPLEMENTARY DATA
- SUPPLEMENTARY DATA
