# Supplementary material for: Simulated binding of transcription factors to active and inactive regions folds human chromosomes into loops, rosettes and topological domains
Source: Nucleic Acids Res. 2016 Apr 8;44(8):3503–12. doi: 10.1093/nar/gkw135 (PMC4856988; doi:10.1093/nar/gkw135)
Supplement: SUPPLEMENTARY DATA [file supp_gkw135_nar-03391-h-2015-File008.pdf]

Simulated binding of transcription factors to active and inactive regions folds human chromosomes into loops, rosettes and topological domains: Supplementary Data

C. A. Brackley[1], J. Johnson[1], S. Kelly[2], P. R. Cook[3], D. Marenduzzo [1]

[1] SUPA, School of Physics and Astronomy, University of Edinburgh, Peter Guthrie Tait Road, Edinburgh, EH9 3FD, UK

[2] Department of Plant Sciences, University of Oxford, South Parks Road, Oxford OX1 3RB, UK,

[3]Dunn School of Pathology, University of Oxford, South Parks Road, Oxford OX1 3RE, UK

(Dated:)

I. Coarse grained molecular dynamics simulations

II. Force fields

III. Mapping simulation units to physical units

IV. Additional simulation details

V. Initialization

VI. Analysing contacts: contact maps, boundaries and rosettograms

VII. Analysis of bioinformatic data

VIII. Supplementary Figures

IX. Supplementary Table

X. Supplementary Movie Captions

XI. References

I. COARSE GRAINED MOLECULAR DYNAMICS SIMULATIONS

In our coarse grained molecular dynamics simulations, we represented chromatin as a bead-and-spring chain, and protein complexes as additional beads. The position of the ith bead in the system changes in time according to the Langevin equation

$$m_i \frac{d^2\mathbf{r}_i}{dt^2} = -\nabla U_i - \gamma_i \frac{d\mathbf{r}_i}{dt} + \sqrt{2k_BT\gamma_i}\boldsymbol{\eta}_i(t), \qquad \text{(S1)}$$

where $\mathbf{r}_i$ is the position of bead i, m_i is its mass, γ_i is the friction it feels due to an implicit aqueous solvent, while $\boldsymbol{\eta}_i$ is a vector representing random uncorrelated noise which obeys the following relations

$$\langle \eta_\alpha(t)\rangle = 0 \text{ and } \langle \eta_\alpha(t)\eta_\beta(t')\rangle = \delta_{\alpha\beta}\delta(t-t'). \qquad \text{(S2)}$$

The noise is scaled by the energy of the system, given by the Boltzmann factor k_B multiplied by the temperature of the system T, taken to be 310 K for a cell. The potential U_i is a sum of interactions between bead i and all other beads, and we use phenomenological force fields as described below. For simplicity we assume that all beads in the system have the same mass and friction $m_i \equiv m$, and $\gamma_i \equiv \gamma$. Eq. (S1) is solved in LAMMPS using a standard Velocity-Verlet algorithm.

II. FORCE FIELDS

For the chromatin fiber the ith bead in the chain is connected to the $i+1$th with a finitely extensible non-linear elastic (FENE) spring: the associated potential is given by

$$U_{\rm FENE}(r_{i,i+1}) = U_{\rm WCA}(r_{i,i+1}) - \frac{K_{\rm FENE}R_0^2}{2}\log\left[1-\left(\frac{r_{i,i+1}}{R_0}\right)^2\right], \qquad \text{(S3)}$$

where $r_{i,i+1} = |\mathbf{r}_i - \mathbf{r}_{i+1}|$ is the separation of the beads, and the first term is the Weeks-Chandler-Andersen (WCA)

potential

$$\frac{U_{\rm WCA}(r_{ij})}{k_BT} = \begin{cases} 4\left[\left(\frac{d_{ij}}{r_{ij}}\right)^{12} - \left(\frac{d_{ij}}{r_{ij}}\right)^{6}\right] + 1, & r_{ij} < 2^{1/6}d_{ij} \\ 0, & \text{otherwise,} \end{cases} \tag{S4}$$

which represents a hard sphere-like steric interaction preventing adjacent beads from overlapping. In Eq. (S4) d_{ij} is the mean of the diameters of beads i and j. The diameter of the chromatin beads is a natural length scale with which to parametrize the system; we denote this by σ, and use this to measure all other length scales. The second term in Eq. (S3) gives the maximum extension of the bond, R_0; throughout this work we use $R_0 = 1.6\,\sigma$, and set the bond energy $K_{\rm FENE} = 30\,k_BT$.

The bending rigidity of the polymer is introduced via a Kratky-Porod potential for every three adjacent DNA beads

$$U_{\rm BEND}(\theta) = K_{\rm BEND}\left[1 - \cos(\theta)\right], \tag{S5}$$

where θ is the angle between the three beads as give by

$$\cos(\theta) = [\mathbf{r}_i - \mathbf{r}_{i-1}] \cdot [\mathbf{r}_{i+1} - \mathbf{r}_i], \tag{S6}$$

and $K_{\rm BEND}$ is the bending energy. The persistence length in units of σ is given by $l_p = K_{\rm BEND}/k_BT$.

Finally, steric interactions between non-adjacent DNA beads are also given by the WCA potential [Eq. (S4)]. In the absence of proteins, the force field of chromatin is therefore appropriate for a biopolymer in a good solvent.

Each protein (or protein complex) we simulate is represented by a single bead; unless otherwise stated, the WCA potential is used to model steric interactions between these. Chromatin beads are labeled as binding or not-binding for each protein species according to the input data. For the interaction between proteins and the chromatin beads labeled as binding, we use a shifted, truncated Lennard-Jones potential, whose form is given by

$$U_{\rm LJcut}(r_{ij}) = \begin{cases} U_{\rm LJ0}(r_{ij}) - U_{\rm LJ0}(r_{\rm cut}) & r_{ij} < r_{\rm cut}, \\ 0 & \text{otherwise,} \end{cases} \tag{S7}$$

with

$$U_{\rm LJ0}(r) = 4\epsilon'\left[\left(\frac{d_{ij}}{r}\right)^{12} - \left(\frac{d_{ij}}{r}\right)^{6}\right],$$

where $r_{\rm cut}$ is a cut off distance, and r_{ij} and d_{ij} are the separation and mean diameter of the two beads respectively. This leads to an attraction between a protein and a chromatin bead if their centres are within a distance $r_{\rm cut}$. Here ϵ' is an energy scale, but due to the second term in Eq. (S7) this is not the same as the minimum of the potential, which for clarity we denote as ϵ (and we refer this to as the interaction energy). For simplicity we set the diameter of the protein complexes equal to that of the chromatin beads, $d_{ij} = \sigma$, and set $r_{\rm cut} = 1.8\,\sigma$ unless otherwise stated.

The length scale σ, mass m and energy scale k_BT give rise to a natural simulation time unit $\tau_{\rm LJ} = \sqrt{\sigma^2 m/k_BT}$, and Eq. (S1) is integrated with a constant time step $\Delta t = 0.01\tau_{\rm LJ}$, for a total of 6×10^6 time steps or more (see main text).

III. MAPPING SIMULATION UNITS TO PHYSICAL UNITS

In order to compare simulation and experimental time and length scales, it is useful here to describe how to map simulation into physical units (this is not required for energy as this was previously expressed in units of k_BT).

Length scales are easily mapped once the value of σ is set in physical units. For simulations of chromatin fibers where one bead corresponds to 3 kbp, a natural choice is σ =30 nm, leading to a linear baseline packing of 10 nm/kbp. For the higher resolution simulations of the chr12 and chr6 regions (Figs. 4 and S12), σ corresponds to 1 kbp. Assuming the same chromatin density in the two models, a unit of length now corresponds to 20.8 nm.

In order to map time units, we need to recognise that there are three main time scales in the system. One is the previously defined Lennard-Jones time τ_{LJ}. A second is the inertial time $\tau_{\rm in} = m/\gamma_i$ (from Eq. (S1)), which is the characteristic time over which a bead loses information about its velocity. A third typical time is the so-called Brownian time $\tau_{\rm B} = \sigma^2/D_i$, which gives the order of magnitude of the time it takes for a bead to diffuse across its own diameter σ. Here D_i is the diffusion constant for bead i, given through the Einstein relation by $D_i = k_BT/\gamma_i$. If we make the approximation that a chromatin bead will diffuse like a sphere we can then use Stokes' law, where $\gamma_i = 3\pi\nu d_i$, with ν the viscosity of the fluid, and d_i the diameter of bead i. Taking realistic values for the length, mass and viscosity one finds that $\tau_{\rm in} \ll \tau_{\rm LJ} \ll \tau_{\rm B}$, with the times separated by several orders of magnitude. For numerical stability we must choose the time step Δt smaller than all of these times, and we wish to study phenomena which will occur on times of the order $\tau_{\rm B}$; this means that using real values for all parameters would lead to unfeasibly long run times. Instead we chose parameters such that $\tau_{\rm in} \le \tau_{\rm LJ} \le \tau_{\rm B}$, and map from simulation to physical time scales through the Brownian time $\tau_{\rm B}$. This assumption means that processes which occur on time-scales below the Brownian time are not resolved accurately, however this is of no practical consequence for our work as we are interested in time-scales much exceeding the Brownian time.

For simulations where chromatin beads were 30 nm in diameter (all except Figs. 4 and S12), taking a viscosity of 10 cP for the nucleoplasm (10 times that of water, to account for the effective increase in viscosity due to crowding) gives a Brownian time of about 0.6 ms, so that a simulation run of 5×10^6 time steps corresponds to about 30 s of real time. For simulations where chromatin beads correspond to 1 kbp (20.8 nm in diameter), one simulation unit of time (one Brownian time) corresponds to about 0.2 ms.

IV. ADDITIONAL SIMULATION DETAILS

For the simulation in Figures 3D and 3E, the force field discussed in Section II was supplemented with torsional interactions to generate results for loops with linking number Lk equal to 0 or 32. To model supercoiled or torsionally relaxed (but not nicked) loops, we use closed

loops (each of contour length 324 σ), which were joined to a linear backbone with a Gaussian spring. We modeled torsional interactions using spherical atoms with an associated triad of vectors, so that the Euler angles describing the relative orientation of adjacent beads allow us to track the twist as well as the bending rigidity. This scheme corresponds to model 2 described in Ref. (1); chromatin was modeled as a ribbon in the torsionally relaxed state, and with torsional persistence length equal to 20 σ.

For convenience, we also list here Lennard-Jones parameters for all attractive interactions in the simulations (the rest of the interactions are repulsive and modeled using a WCA potential, as previously mentioned). The interaction range (cut-off of Lennard-Jones interaction) was equal to 1.8 σ for all attractive interactions. Interaction strengths (ϵ', in units of k_BT) were as follows: Figures 1, 3A, 3B and S2B: 7.1 (between red "transcription factors" and pink beads); 3.5 (between red factors and blue beads). Figure 2A: 8.9 (between red factors and pink beads; and between green factors and light-green beads). Figures 2C and S5: 7.1 (between each of the factors and its target binding beads). Figures 3C: 3.5 (between red factors and pink beads; and between green factors and light-green beads). Figures 3D and 3E: 3.5 (between red factors and pink beads. Figures 4, 5 and S12): 7.1 (between red factors and pink beads); 3.5 (between red factors and light-green beads; and between black "proteins" and grey beads). Figure S2A: 7.1 (between red factors and pink beads). Figure S3: 7.1 (between either red or green factors and yellow beads). Figure S4: 7.1 (between red factors and pink beads; and between green factors and light-green beads). Figure S6: initially 7.1 only between red factors and pink beads; after "switch" 7.1 (between red factors and pink beads), and 13.1 (between green factors and light-green beads).

V. INITIALIZATION

Finally, as in all molecular dynamics simulations it is important to specify how the system was initialised. For all cases where a single linear polymer was modeled, chromatin fibers were first generated as random walks, and proteins randomly distributed (with uniform probability throughout the simulation box). The simulation was then run with a soft potential between all beads to remove overlaps, and with a Gaussian spring between neighboring beads (this was for at least a million time steps; in some cases it was also necessary to use a higher bending rigidity to avoid initial entanglements). After equilibration, the force field was set to the one discussed in Section II. For Figures 3E and 3F, we first equilibrated supercoiled or torsionally relaxed loops in isolation, then joined them to a linear backbone at appropriate places (see caption to Fig. 3) with Gaussian springs; the system was then allowed to equilibrate with the force field in Section II (which preserves topology and linking number as it disallows intrachain crossings). For Figure S5, we generated initial conformations for the 20 chromatin fibers as mitotic-like cylinders with random orientation, following the method described in Ref. (2); proteins were still distributed randomly and uniformly at the beginning of the simulations. The equilibration steps were then performed as above (with soft potential and Gaussian springs).

VI. ANALYSING CONTACTS: CONTACT MAPS, BOUNDARIES AND ROSETTOGRAMS

An important output of both Hi-C experiments and our simulations are contact maps; in this Section we discuss how we analysed them.

The contact maps in Figures 1D, 2Aiii, S2Aiii, S2Biii, S3iii, S4iii, S5iii and S7F were obtained from a single configuration: a contact between two beads was scored if their centers were less than 150 nm (5 σ) apart. We binned contacts by dividing the polymer into a number of bins (specified in each Figure Legends) to aid visualization. The colored contact maps in Figures 2Aiii, S3iii, S4iii, S5iii and S6iv were also obtained from a single configuration, by only considering the binding sites. A contact between two binding sites was scored if their centers were less than 90 nm (3 σ) apart. Binding sites were colored according to the protein (or factor) which is attracted to them (i.e., pink sites are colored red as they bind red factors, etc.); in case a binding site could be the target for more than one factor (e.g., in Fig. S6iv), we colored the binding site according to the protein which was closest to them (e.g., red if the binding site was closest to a red protein, etc.). Pixels in the contact map then are colored red if they are contacts between two red pixels, etc.; mixed contacts are colored yellow if between red and green, and grey in Figure S5iii. Finally, the simulation contact maps in Figures 3, 4, 5, S7A-E and S12, were averaged over several realizations (specified in the Figure Legends, together with the binning used). In all contacts map (with the exception of colored contact maps), the entry gives the number of contacts in the bin, scaled by the maximum number of contact maps over all bins (in this way entries are between 0 and 1).

For each of the simulations in Figure 3, we plotted both the whole contact map (Fig. S7) and just the part of it close to the diagonal (referred to as pyramid plots in the text); the latter is often used in the literature as it allows a clearer visual determination of boundaries. While the simulated contact maps are shown without any normalization, experimental contact maps for GM12878 cells (Figs. 3 and 5 in the main text) were normalized according to the square root normalization method described in Ref. (3). Experimental contact maps for Figure S12 were not normalized; these were computed from the Sequence Read Archives (SRA) data in the Gene Expression Omnibus [obtained from Ref. (4) via access number GSE35156; duplicate reads were removed].

For each of the contact maps (whether from simulation or experiments), we prepared Janus and difference plots, and computed the number of contacts (or contact probability) versus distance along the genome/simulated chromatin fibers. All contact maps were binned (the binning used varied in the different cases and in specified in Figure Legends).

The Janus forward signal for bin i, $F(i)$, is defined as

the sum over all contact map entries relative to contacts which a bead makes with other beads to the right of it: i.e., $\sum_{j=i+1}^{n} c(i,j)$, where $c(i,j)$ denotes the contact map entry relative to the i-th and $j-$th bins, and n is the total number of bins. The Janus backward signal for bin i, $B(i)$, was similarly computed as $\sum_{j=1}^{i-1} c(i,j)$.

The difference plot (Figs. S8 and S9) is the difference $\Delta(i) \equiv F(i) - B(i)$. When this quantity is negative, bin i is making more contact to its left; when it is positive, the majority of the contacts bin i makes are to its right. The difference plot is useful to get a first estimate of domain boundary locations, since boundaries are places where the pattern of contacts made by a bin changes from mostly to the left to mostly to the right (but not vice versa). Therefore, boundaries can be located at regions where $\Delta(i)$ crosses 0 with an upward derivative; a similar algorithm was used to locate boundaries in Ref. (4). This is the base of the algorithm used in Figure S9 to determine boundaries automatically in the region chr12:85000000-100000000 bp (Fig. 4 in the main text). To avoid spurious multiple nearby boundaries due to noise in the difference plot signal, we further required that either the upward trend in $\Delta(i)$ is common to 4 consecutive beads crossing zero, or that the upward slope at the zero crossing (forward difference $\Delta(i+1) - \Delta(i)$ where $\Delta(i) < 0$ and $\Delta(i+1) > 0$) is larger than a set threshold (equal to 10% or 40% of the maximum step in the function $\Delta(i)$, for experiments and simulations respectively).

Another way to detect boundaries is via peaks in the derivative of $\Delta(i)$ (this is the insulator plot in Fig. S8): the rationale here is that we expect the relative fraction of contacts to the right should increase sharply at boundaries, however due to contacts away from the diagonal it may not necessary be that the difference plot goes through 0. In selected cases we also used an adaptation of the recent method described in Ref. (5) to detect boundaries. While all methods agreed on some of the boundaries, visual inspection suggests that not all boundaries can be found by any one automated technique (see Figs. 4 and S11B). While in Hi-C experiments the numerical error that these or similar algorithms make is not too important, it is much more consequential with simulation data that are noisier. Moreover, our goal is to compare experimental and simulation boundaries, rather than to estimate boundaries in either simulations or experiments with a given accuracy. Comparing boundaries in simulations and Hi-C data is a demanding task: for instance, even a two pixel error (the $\Delta(i)$ curve turning in an opposite direction) would lead to an artificial discrepancy of 80 kbp between the location of the same boundary in simulations and experiments, and missing out boundaries or false detection of boundaries due to noise would give an even more serious reduction in the measure of the agreement between simulations and experiments (as the number of boundaries which can be located randomly is relatively high, see text). As a result, while the automated detection of boundaries in Figure S9 shows that the agreement between simulations and experiments is statistically significant, there are errors in the boundary detection which affect this comparison. To avoid this, we resorted to locating boundaries by visual inspection (compare Fig. 4 with Fig. S9).

Finally, we discuss some details of rosettograms (Fig. S1B). To build these, we start from one configuration from the simulation, and divide the binding beads into clusters; two binding beads are defined to be in the same cluster if their separation is below a threshold (typically 90 nm, unless specified otherwise). Then clusters are numbered, starting from the first along the chromatin fiber (Fig. S1B). The rosettogram plots cluster number versus binding bead number, and for clarity we only show the binding beads which are in clusters. A string of well formed rosettes shows up as a series of continuous lines (lines made up by contiguous pixels) in the rosettogram, whereas a more disordered structure with lots of non-local contacts is characterised by breaks in the horizontal lines in the rosettogram (as binding beads in the cluster will often not be contiguous along the polymer chain). To quantify how disordered (i.e., how far from an ideal string of rosettes) the loop network of a chromatin fiber is, we compute the *fraction disorganised*, or f_d. To define f_d, we count the number of steps (upwards or downwards) in the rosettogram. In an ideal string of N rosettes there will be $N-1$ steps, so if N denotes cluster number, we subtract $N-1$ from the number of steps: this gives the number of "errors", i.e., of non-local loops in the interaction network. The fraction disorganised is then defined as the number of errors per pixel (i.e., the number of errors divided by the number of pixels in the rosettogram). From this definition, it is apparent that a small value of f_d indicates a structure akin to a regular string of rosettes, whereas a large value indicates a disordered structure with many non-local contacts.

VII. ANALYSIS OF BIOINFORMATIC DATA

Here, we explain how beads were colored using bioinformatic data in the chromosome simulations (Fig. 4, for chr12:85000000-100000000 bp, Fig. S12 for chr6:5000000-20000000 bp, and Fig. 5 for the whole of chr19).

Beads in the simulations can interact either with (black) "proteins" binding to heterochromatin (when they are colored grey) or with (red) "transcription factors" binging to euchromatin (when they are colored pink or light-green according to binding affinity), or with both (indicated in cartoons by the surrounding halo), or with neither (when they are colored blue).

Data from the Broad ChromHMM track on the hg19 assembly of the UCSC Genome browser were used to determine pink/light green coloring as follows: (i) if a region of 90 bp or more within one bead (representing 1 kbp, Figs. 4 and S12, or 3 kbp, Fig. 5) is labeled as an "Active Promoter" or "Strong Enhancer" (states 1,4,5 on the Broad ChromHMM track), then the whole bead is colored pink (so it binds with high affinity to red factors representing transcription factor/polymerase complexes); (ii) if a region of 90 bp or more within the sequence covered by one bead is labeled "Transcriptional Transition" or "Transcriptional Elongation" (states 9 and 10), then that bead is colored light green (and binds red factors with low affinity).

To determine whether a bead should be colored grey (i.e., labeled as heterochromatin) we used one of the fol-

lowing two methods. Either we directly used the Broad ChromHMM data (in Fig. S12): if 90 bp or more within the bead is classified as state 13, then the whole bead is classified as grey. Alternatively (in Figs. 4, 5 and S12) GC content data from the UCSC Genome Browser were used to color beads by setting a threshold GC content percentage and coloring beads grey if they fell below this. Here, the rationale behind this is that heterochromatin and gene poor regions are known to correlate with low GC content (they are rich in AT). The threshold was set, in each case, so as to end up with the same overall number of heterochromatic beads as one would obtain if beads were colored grey according to the Broad ChromHMM track. As a result, the %GC content threshold used was 43.4% for chr6, 41.8% for chr12 and 48.4% for chr19. For chr19, some of the telomeric sequences are missing for hg19; we have assumed these are not binding to black proteins. We note that our coloring scheme (both when only using the HMM track and when also using GC content) allows a bead to be of more than one color. This is sensible in view of our coarse graining (a single bead can include both a euchromatic and a heterchromatic region), and also become some genomic regions can be targets for competing chromatin-associating proteins.

VIII. SUPPLEMENTARY FIGURES

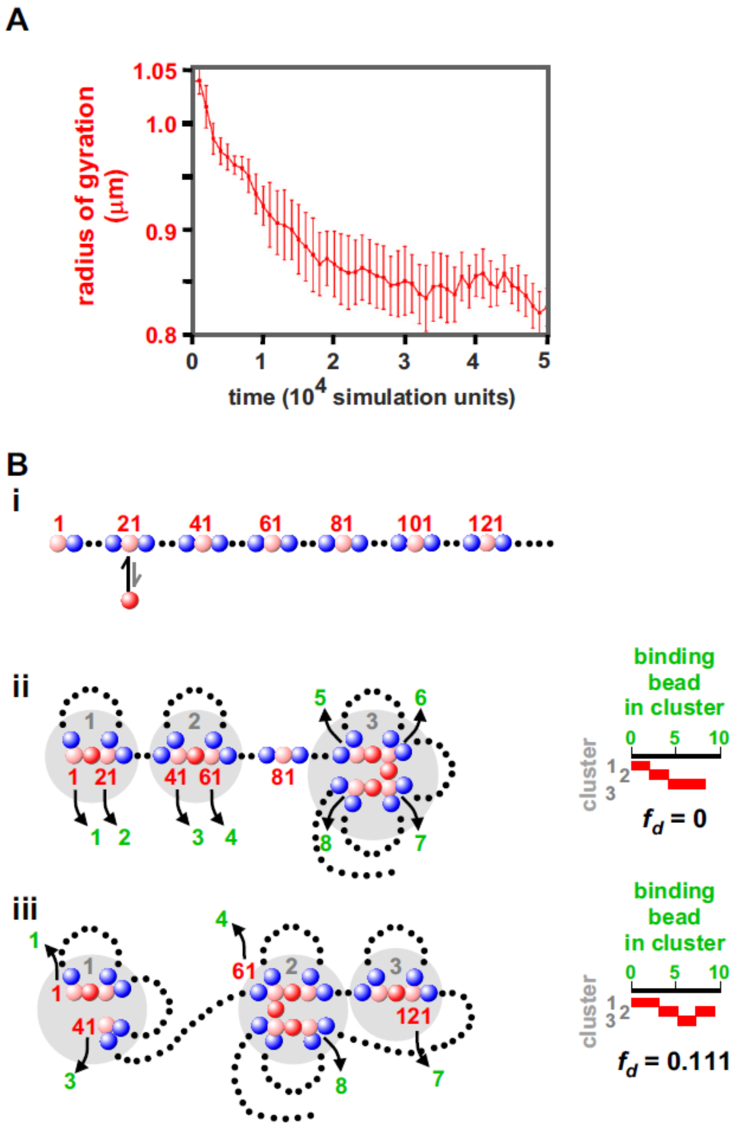

Figure S1. Equilibration and example "rosettograms". (A) Plot of the radius of gyration as a function of time (after switching on chromatin:protein binding). The gyration of radius is in steady state, or changes very little, by the end of the simulation. [Errors: standard deviations of the mean.] (B) Example "rosettograms". (i) Factors (red) can bind to every 20th bead (pink) in the fiber. (ii,iii) Two possible structures (left), and corresponding rosettograms (right). (ii) A simple structure used to illustrate the numbering system. First, clusters (grey circles) are defined (a cluster contains $\geq$2 binding beads with centers lying <90 nm apart). Clusters are then numbered from 1 upwards (grey numbers), beginning with the one containing the lowest- numbered binding bead in the fiber. Binding beads in clusters (but not blue beads, or bead 81 – which is not in a cluster) are now renumbered as shown in green. In a rosettogram, a red pixel marks the presence of a binding bead in a cluster. In a row, increasing numbers of abutting (conversely, non-abutting) pixels reflect increasing numbers of near neighbor (conversely, distant neighbor) binding beads in a rosette and an organized (conversely, disorganized) structure. The disorganized fraction (f_d) is equal to $(S - N + 1)/P$, where S is the number of steps in the rosettogram, N is the number of clusters (or rows), and P is the total number of colored pixels (i.e., the total number of binding beads, see Supplementary Information for a motivation for this formula and for a further discussion of f_d). This quantity is also equal to the total number of white spaces between first and last colored pixels in each row divided by P (i.e., the total number of binding beads in clusters). Here, f_d is 0 (the low value reflects an ordered structure where all loops involve nearest-neighbor binding-sites). (iii) A more complicated structure gives a more complex rosettogram with non-abutting pixels in row 2; as there is one gap between red pixels in row 2 and 9 pixels in all, $f_d = 1/9$.

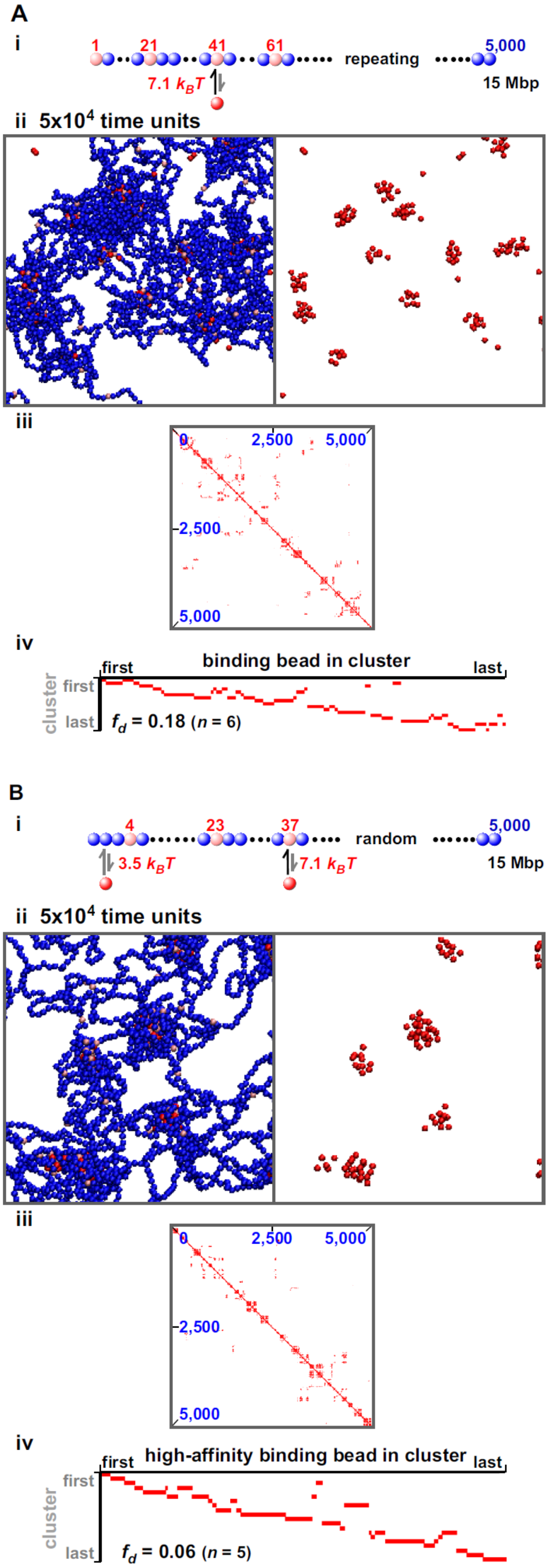
A
i
1
21
41
61
repeating
5,000
7.1 kBT
15 Mbp
ii 5x10^4 time units
iii
0
2,500
5,000
2,500
5,000
iv
first
binding bead in cluster
last
cluster
first
last
fd = 0.18 (n = 6)
B
i
4
23
37
random
5,000
3.5 kBT
7.1 kBT
15 Mbp
ii 5x10^4 time units
iii
0
2,500
5,000
2,500
5,000
iv
first
high-affinity binding bead in cluster
last
cluster
first
last
fd = 0.06 (n = 5)

Figure S2. Simulations as in Figure 1, showing bound factors still spontaneously cluster if low-affinity binding is absent, or high-affinity sites are randomly distributed. (A) Absence of low-affinity binding. (i) Factors have a high affinity for pink beads, but zero affinity for blue beads. (ii) Final snapshots of a central region with/without chromatin; clusters still form. (iii) Final contact map; blocks along the diagonal are slightly less prominent compared to those seen in Figure 1Aiv (same binning used). (iv) Final rosettogram; most clusters still contain $\geq$2 petals, but runs of abutting pixels in one row are slightly shorter than those seen in Figure 1Avi (and the f_d is higher, indicating a higher-fraction of non-local loops). (B) Randomly-distributed binding sites. (i) Pink beads are distributed randomly along the fiber, with the same average linear density as in Figure 1A. (ii) Final snapshots of a central region with/without chromatin; clusters still form. (iii) Final contact map; blocks along the diagonal are not so uniform and are spaced irregularly. (iv) Final rosettogram. Perhaps surprisingly, the structure is slightly less disorganized than in Figure 1Avi in the main text, and in Figure S2Aiv above (reflected by a lower f_d). This is probably because gaps between successive binding sites are exponentially distributed so that binding sites are naturally clustered nearer together in 1D genomic space ("Poisson clumping"), and this facilitates formation of more "perfect" rosettes containing near-neighbor binding beads.

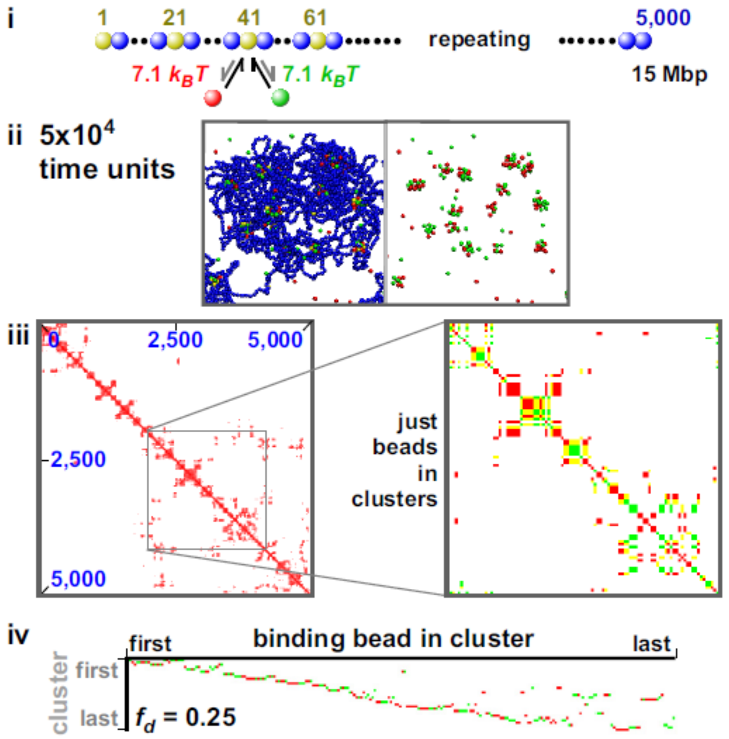

Figure S3. Mixed clusters form if red and green factors can bind to the same high-affinity sites. MD simulations were as in Figure 2A, with the differences indicated. (i) Red (n= 250) and green (n= 250) factors interact solely with every 20th bead (yellow); data below are for the state after 5×10^4 time units. (ii) Final snapshots (with/without chromatin); bound red and green factors are often found in one cluster. (iii) Final contact map for all beads (axes give bead numbers). The zoom shows a high-resolution map of just binding beads in clusters (prepared as in Fig. 2Aiii). Here, red, green and yellow pixels mark contacts between two pink beads (in a cluster and bound to a red protein), between two light-green beads (in a cluster and bound to a green protein), and between a light-green and pink bead, respectively. The many yellow pixels reflect the presence of mixed clusters containing both red and green factors. Note that the patterns of pixels in the regular (left) and high-resolution maps (right) differ slightly both here and in maps shown later; this is the result of the different criteria used to define contacts, and whether binning was used. (iv) Rosettogram (pixels colored according to which high-affinity beads are in the cluster). Rows often contain contiguous pixels of different colors, again reflecting the presence of both types of factor in one cluster. Intriguingly, the value of f_d is higher than that seen with the distinct clusters in Figure S2iv, presumably, this is due to the higher number of proteins in the simulation (500 as opposed to 250).

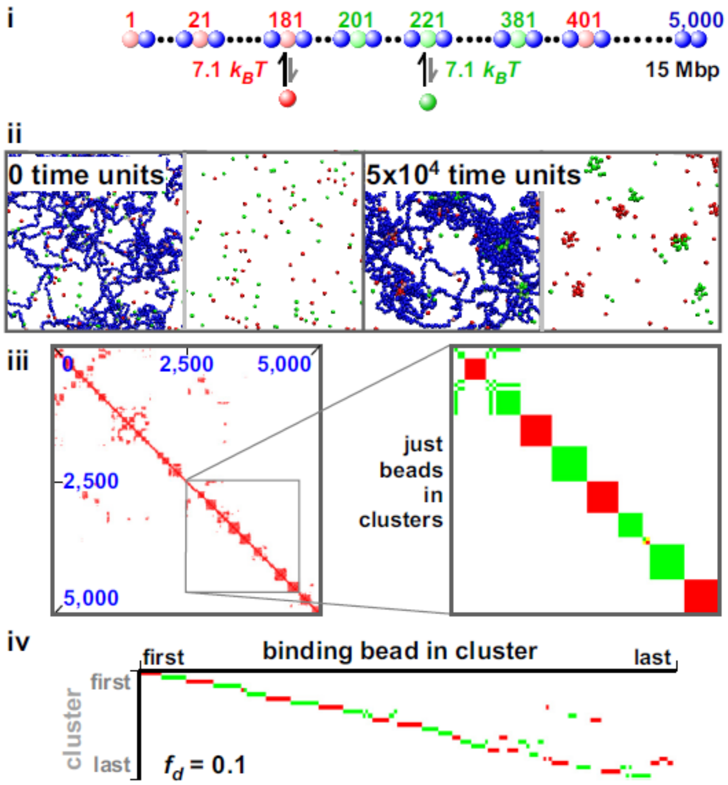

Figure S4. Red and green proteins form distinct clusters if their cognate sites are present in distinct blocks (mimicking eu- and hetero-chromatin). Conditions as in Figure 1, with exceptions indicated. (i) Red (n= 250) and green (n= 250) factors interact solely with pink and light-green beads, respectively; 10 pink and 10 light-green beads are found at every 20th position from beads to 1-181 and 201-381, respectively, and this pattern repeats. Data given below are for the state after 5x10^4 time units. (ii) Snapshots (with/without chromatin); red and green factors are found in distinct clusters. (iii) Contact map (axes give bead numbers). The zoom shows a high-resolution map of just binding beads in clusters (prepared as in Fig. 2Aiii). Here, red and green pixels mark contacts between two pink beads, or between two light-green beads, respectively. As each block contains 10 binding beads (just less than the $\sim$12 typically found in a cluster in Fig. 1), as blocks alternate along the fiber, and as the two sets of bound factors assemble into distinct clusters, this fiber folds into a highly-organized structure – which is reflected by the alternating colored squares along the diagonal. (iv) Rosettogram (pixels colored according to which high-affinity beads are in the cluster). Again, this reflects the high level of organization (e.g., some “perfect” rosettes with 10 petals are present, and the f_d is low).

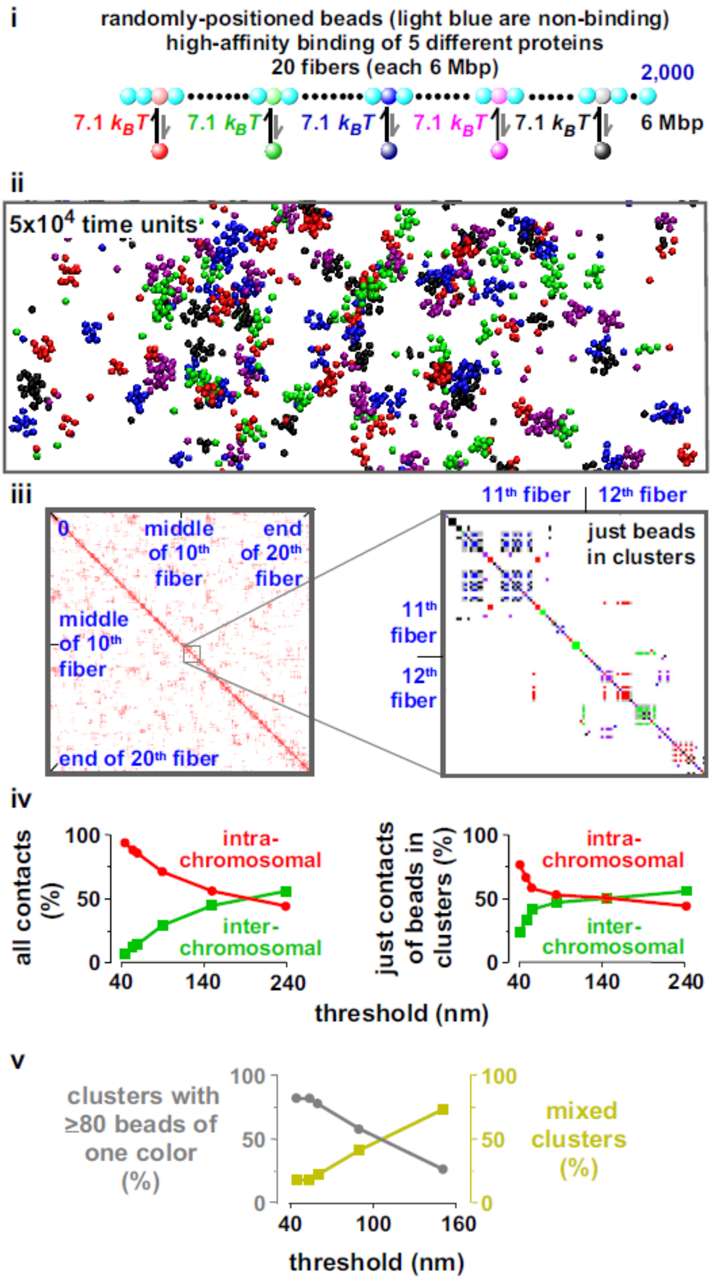

Figure S5. Five different factors form distinct clusters when binding to cognate sites scattered randomly on 20 identical fibers. MD simulations are those of Figure 2C. (i) Red, green, dark-blue, purple, and black factors (500 of each) bind (7.1 kBT) to five sets of cognate sites scattered randomly along 20 identical fibers (each with 2,000 beads representing 6 Mbp). [Randomly scattering binding sites so that one in 20 beads can bind a factor led in this case to 381, 385, 383, 437, and 416 binding beads in total for red, green, dark-blue, purple, and black factors, respectively.] Data presented below were obtained after 5x10^4 time units. (ii) Snapshot (without chromatin for clarity); each factor tends to cluster with others of the same color (the center of this image is presented in Fig. 2C). (iii) Contact map for all beads in every fiber (axes show positions; contacts made by every 100 adjacent beads on a fiber are binned). The zoom shows a high-resolution map of just binding beads in clusters (as in Fig. 2Aiii) from bead 715 in fiber 11 to bead 1,123 in fiber 12; grey pixels mark contacts between beads of different colors, and colored ones contacts between two beads of the indicated color. As 47% non-white pixels are grey, most factors are present in clusters that contain only one color. (iv) The effect of the threshold used to define contacts (in nm) on the percentage of intra- and inter-chromosomal contacts between all beads (left), and between just binding beads (right). (v) The effect of the threshold used to define contacts (in nm) on the percentage of clusters in which ≥80% binding beads are of one color, and in the other clusters (mixed).

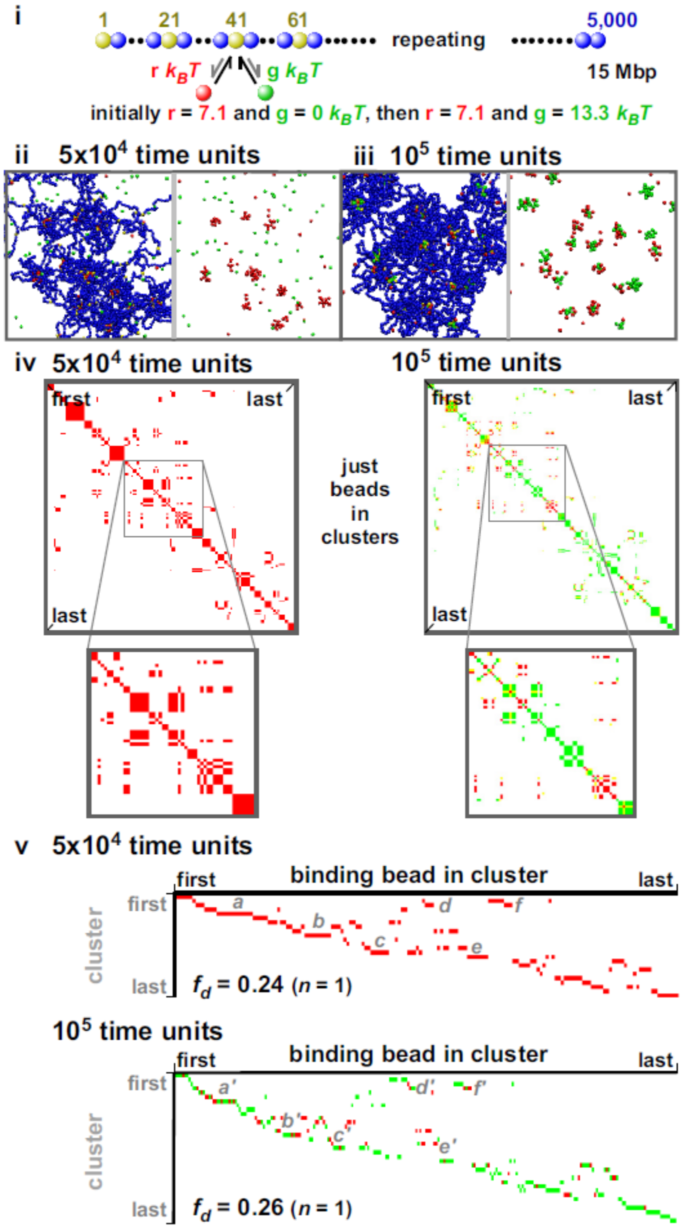

Figure S6. Evolution of one type of cluster into another. Conditions as in Figure 1, with exceptions indicated. (i) Overview. Red and green factors are present (250 of each); yellow beads are found at every 20th position in a 5,000-bead fiber. Initially, red factors interact (7.1 kBT) with yellow beads, but green factors do not interact with any beads. After 5×10^4 time units, green factors acquire affinity (13.3 kBT) for yellow beads (perhaps because they become "phosphorylated"), and the simulation continues for another $5x10^4$ units. (ii,iii) Snapshots (with/without chromatin). The system evolves first into one containing only clusters of bound red factors, and – once green factors start binding with higher affinity red-green (and pure green) clusters develop. (iv) Contact maps of just binding beads in clusters prepared as for the zoom in Figure 2Aiii, where contacts are scored without binning if bead centers lie 90 nm apart, and binding beads are treated as if they possess the color of the nearest factor bound to the fiber. Using this coloring scheme, red, green, and yellow pixels mark contacts between two red beads, between two green beads, and between a green and red bead, respectively. We also show two zooms of a central region of the contact map for binding beads. After 5×10^4 units, only red pixels are seen (as only red factors are bound in clusters, and the green factors are non-binding). After 10^5 units, green pixels predominate. Note that the general patterns seen at the two times are similar; this is because once clusters of red factors appear, the general structure persists after the switch as red factors in a cluster are replaced by green ones. (vi) Rosettograms (pixel corresponds to binding sites, their colors depict those of the nearest bound factor). After $5x10^4$ time units, only red factors are in clusters; after 10^5 units, green pixels predominate with red and green factors sometimes being found in one cluster (giving pixels of different colors in one row). Many clusters also persist from one time to the next (reflected by the pattern of sets of contacts a-f being similar to that of sets a'-f').

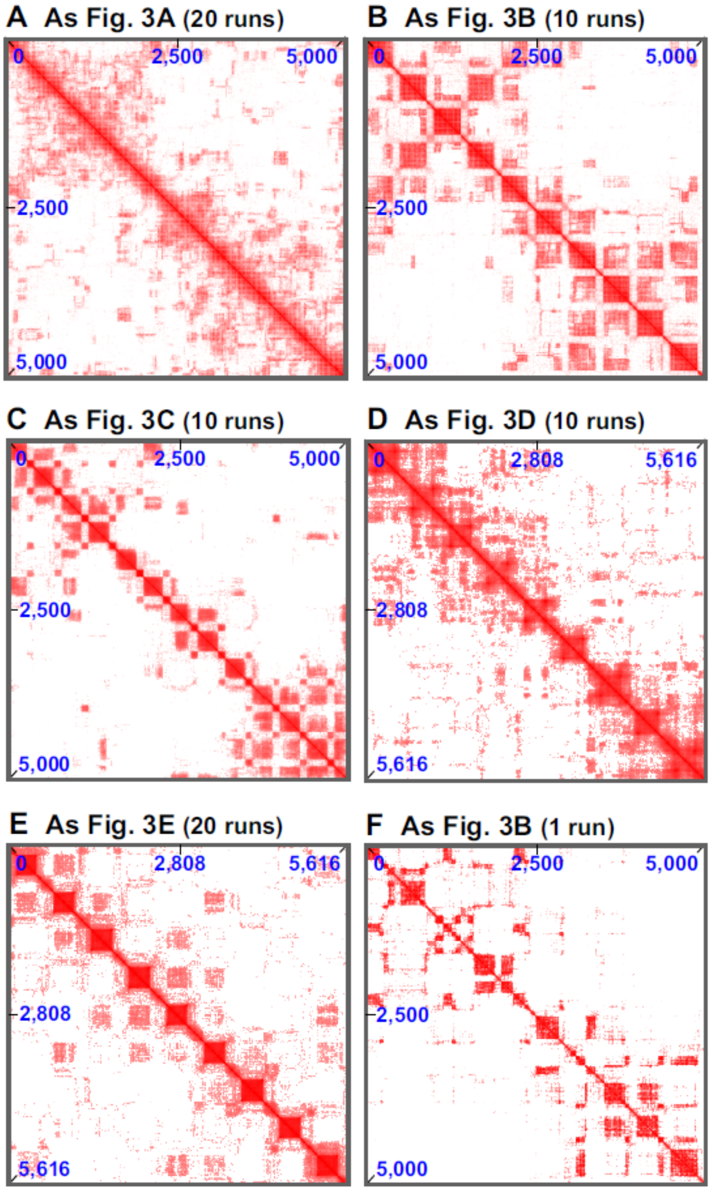

Figure S7. Contact maps supporting Figure 3. (A-E) Truncated contact maps were presented in Figure 3; complete ones are given here. These are averaged over the number of runs indicated. (F) Complete contact map for one run using the conditions in Figure 3B; the off-diagonal blocks (representing inter-domain interactions) visible here contribute only weakly to the population average in (B).

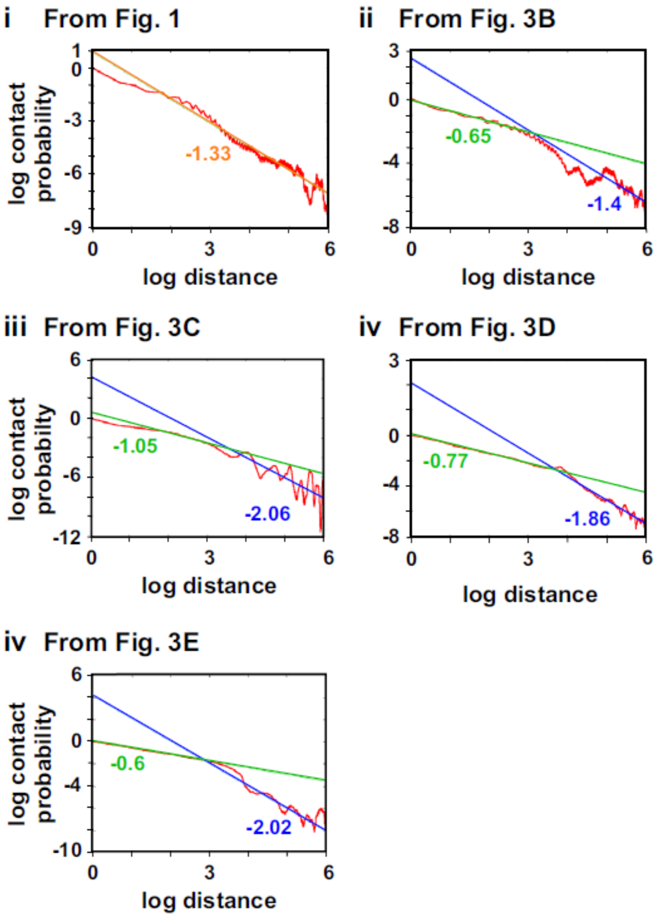

Figure S8. Contact probability as a function of distance (bead number) along the different fibers illustrated in Figure 3 (red curves). Straight lines indicate fits using the exponent indicated (brown line and exponent – fit of entire curve; green line and exponent – fit over short distances and so within a domain; blue lines and exponents – fits over longer distances and so between domains). The effective exponent depends on distance (intra-domain versus inter-domain) and conditions; similar conclusions were reached by Barbieri *et al.* (2009). Here “log” denotes the natural logarithm; distances are measured in units of bead size.

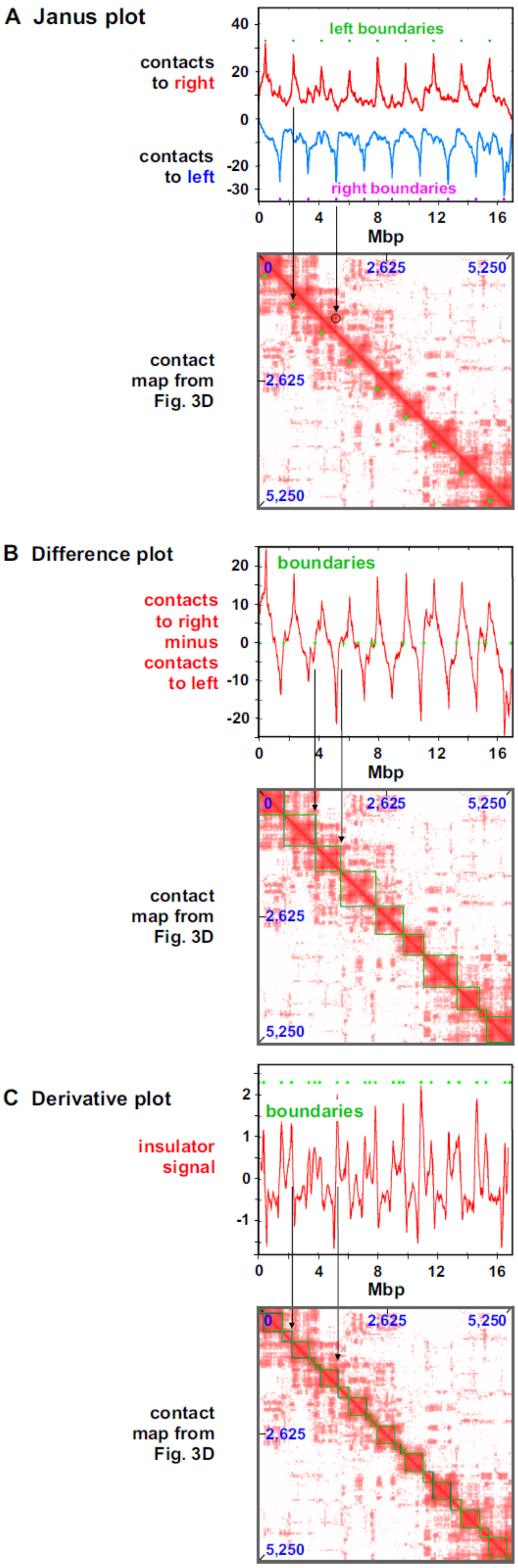
A Janus plot
contacts to right
contacts to left
left boundaries
right boundaries
40
20
0
-20
-30
0
4
8
12
16
Mbp
contact map from Fig. 3D
0
2,625
5,250
B Difference plot
contacts to right minus contacts to left
boundaries
20
10
0
-10
-20
Mbp
contact map from Fig. 3D
C Derivative plot
insulator signal
boundaries
2
1
0
-1
Mbp
contact map from Fig. 3D

Figure S9. Identifying loops and boundaries in contact maps. This Figure shows examples of how loops and boundaries can be identified using data in the contact map in Figure 3D (reproduced in each case below each of the 3 plots, with vertical arrows indicating related positions). (A) "Janus" plot. This signal is proportional to the number of contacts made by each bead to the right (red curve) and to the left (blue curve). In the top plot (red), green circles identify peaks; these mark beads at the left boundary of each loop. In the bottom plot (blue), purple circles identify the bottom of valleys; these mark beads at the right boundary of each loop. The coordinates of the left and right tethers found in this way can be used to identify contacts corresponding to the base of the loop (these are shown as green dots or circles in the contact map). (B) Difference plot. This shows the difference in contacts made by every bead (binned, with 7 beads/bin) to the right and to the left (i.e., the blue curve in A is subtracted from the red curve in A, see Supplementary Information; this plot is analogous to the one used by Dixon *et al.*, 2012). When the plot intersects zero with an upward derivative, it means that the pattern of contacts switches from contacts mainly to the left to mainly to the right (the behavior expected of a boundary). (C) Derivative plot (see Supplementary Information, and Dixon *et al.*, 2012) of data in (B). This can be viewed as a plot of an "insulator" signal, as now boundaries are identified with peaks (i.e., regions where the pattern of contact changes abruptly over a short genomic region).

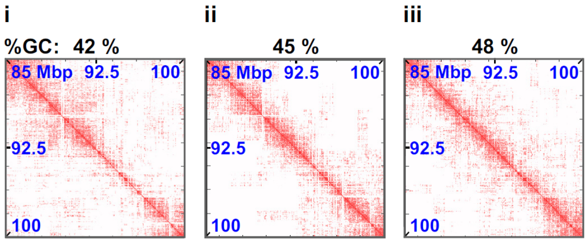

Figure S10. Effect of % GC threshold on contact maps (chr 12, GM12878 cells). Average contact maps (over 10 runs) for the same region of chr12 simulated in Figure 4, but where the threshold to define a bead as heterochromatic is modified. In Figure 4, the threshold was 41.8 %; these plots show that the contact map for thresholds of 42, 45 and 48 % respectively. The contact map is very similar for (i) and (ii); in (iii) the GC threshold is large enough that some active regions are labelled as heterochromatin, which results in fuzzier domains.

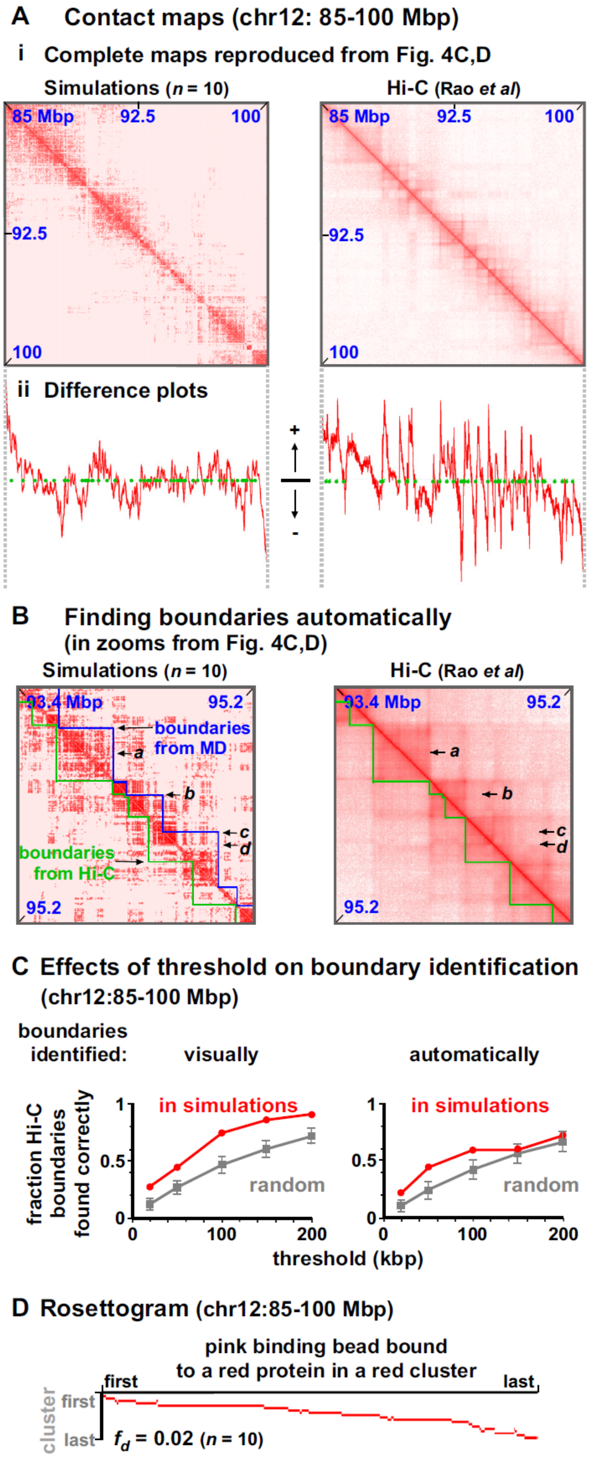
A Contact maps (chr12: 85-100 Mbp)
i Complete maps reproduced from Fig. 4C,D
Simulations (n = 10)
Hi-C (Rao et al)
85 Mbp
92.5
100
ii Difference plots
+
-
B Finding boundaries automatically
(in zooms from Fig. 4C,D)
Simulations (n = 10)
Hi-C (Rao et al)
93.4 Mbp
95.2
boundaries from MD
boundaries from Hi-C
a
b
c
d
C Effects of threshold on boundary identification
(chr12:85-100 Mbp)
boundaries identified:
visually
automatically
fraction Hi-C boundaries found correctly
in simulations
random
1
0.5
0
100
200
threshold (kbp)
D Rosettogram (chr12:85-100 Mbp)
pink binding bead bound
to a red protein in a red cluster
first
last
cluster
f_d = 0.02 (n = 10)

Figure S11. Different ways of identifying boundaries in 15 Mbp on chromosome 12 (in GM12874) and the associated rosettogram. Simulations were those used for Figure 4. (A) Contact maps and difference plots. (i) Contact maps are reproduced from Figures 4C,D. (ii) Plot of the difference between contacts to the right and left for a given location on the simulated fiber (left; 7 kbp/bin) or the chromosome (right; 10 kbp/bin). Boundaries found by visually inspecting the contact maps are shown by green dots; many (especially those close to the boundaries of the region analysed) but not all lie at or close to points where the plot intersects zero with an upward derivative (plots prepared as in Fig. S9B). (B) Finding boundaries by locating zeros in the difference plot with upward derivatives (see Fig. S9B and Supplementary Information) in the contact maps in Figure 4. Blue and green lines in the zooms (same regions as in Figs. 4C, D) illustrate boundaries found automatically in the simulation and Hi-C data, respectively. Visual inspection indicates the algorithm is only partially successful at identifying boundaries. a: an obvious boundary in the simulation data that is missed by the algorithm (this boundary is also seen in the Hi-C map, but is also missed by the algorithm). b: boundaries detected in both maps, but in the Hi-C map the algorithm places two boundaries very close to each other. c: the algorithm splits an obvious domain (which is seen in the data from simulations). d: another boundary missed by the algorithm in both maps. (C) Effects of threshold on correct prediction of boundaries (determined either by the difference plot aided by visual inspection, or automatically). A boundary is "correctly" predicted if it lies within a distance less than the threshold away from a boundary seen in the Hi-C data. For instance, 27 out of 36 boundaries (a fraction of 0.75) are correctly predicted by the difference plot aided by visual inspection using a 100-kbp threshold. The grey line shows a control plot which gives the fraction of correctly-predicted boundaries found by scattering the same number of boundaries found in a simulation randomly throughout the genomic region analysed. This procedure was repeated 100 times, and error bars in the random control denote the standard deviation. The difference between points on the two curves at most thresholds are highly significant (Table S2). (D). Rosettogram for high-affinity beads in all 15 Mbp. Only pink binding beads that both bind red proteins and are in red clusters are considered. As grey and light-green binding beads are not considered, and as these are often found in long runs, the effects of such long runs on the appearance of rosettograms are minimized; choice of only pink beads that bind red proteins further minimizes the effects of runs of adjacent pink beads. Many red pixels abut in one row, indicating the formation of many rosettes involving nearest-neighbor pink beds. The value of f_d is also low, indicative of many local contacts and an ordered structure.

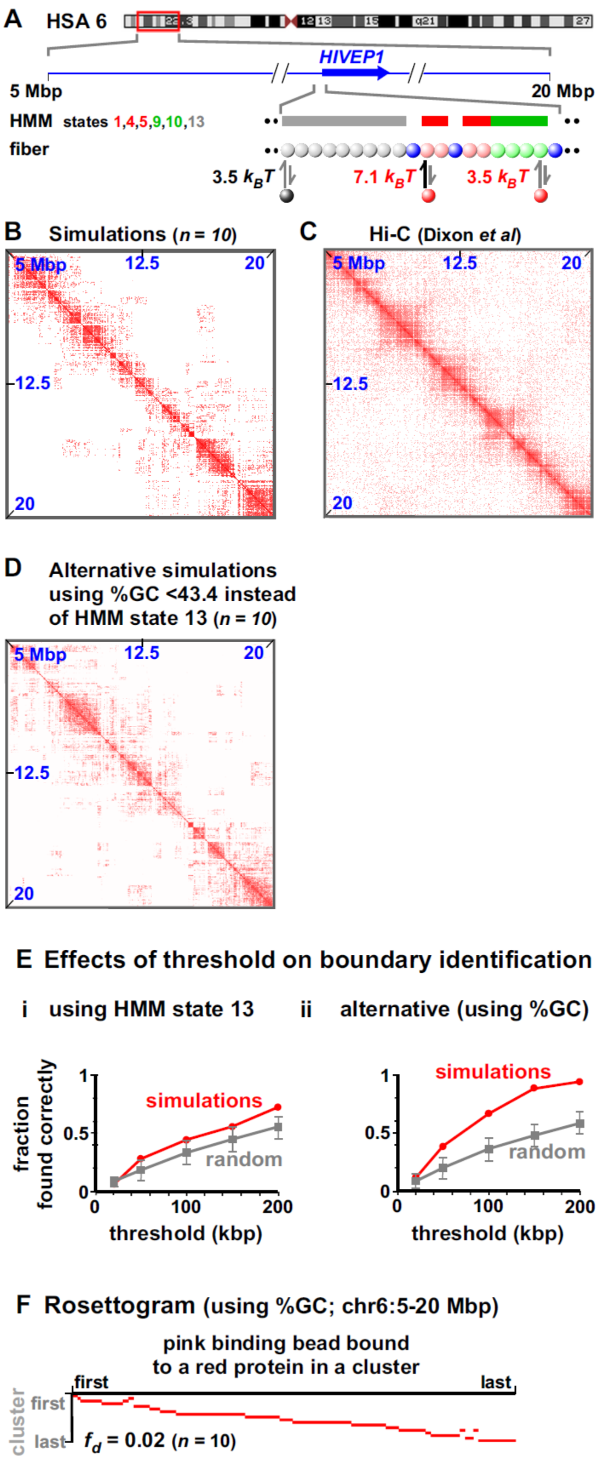
A HSA 6
HIVEP1
5 Mbp
20 Mbp
HMM states 1,4,5,9,10,13
fiber
3.5 k_BT
7.1 k_BT
3.5 k_BT
B Simulations (n = 10)
5 Mbp
12.5
20
C Hi-C (Dixon et al)
D Alternative simulations using %GC <43.4 instead of HMM state 13 (n = 10)
E Effects of threshold on boundary identification
i using HMM state 13
ii alternative (using %GC)
fraction found correctly
simulations
random
threshold (kbp)
F Rosettogram (using %GC; chr6:5-20 Mbp)
pink binding bead bound to a red protein in a cluster
first
last
cluster
f_d = 0.02 (n = 10)

Figure S12. Simulating 15 Mbp of chromosome 6 in H1-hESC cells. (A) Overview. The ideogram (red box gives region analyzed) and HMM track (colored regions reflect chromatin states) are from the UCSC browser; the zoom illustrates the HIVEP1 promoter. Beads (1 kbp) are colored according to HMM state (blue – non-binding, n= 3,890; pink – states 1+4+5, n= 167; light-green states 9+10, n = 723; grey – state 13, n= 10,238). Red factors (n=300) bind to (active) pink and light-green beads with high and low affinities, respectively; black (heterochromatin-binding) proteins (n= 3,000) bind to grey beads. (B,C) Contact maps (7 and 20 kbp binning for simulation and Hi-C data, respectively). (D) Contact map obtained using alternative simulations, in which grey beads were selected using %GC >43.4 (instead of HMM state 13). [This %GC gives the same number of grey beads as the use of HMM state 13.] The overall pattern is similar to that seen in (B). (E) Effects of threshold on correct prediction of boundaries (determined by difference plots aided by visual inspection). A boundary is "correctly" predicted if it lies within a distance less than the threshold away from a boundary seen in the Hi-C data. The grey line shows a control plot which gives the fraction of correctly-predicted boundaries ($\pm$ SD) found by scattering randomly the same number of boundaries found in a simulation throughout the genomic region analysed. (i) Results obtained using the simulation illustrated in (A) and the contact map in (B). (ii) Results obtained using the alternative set of simulations that give the contact map in (D), and a higher fraction of correctly-identified boundaries. The difference between points on the two curves at each threshold are now highly significant (see Table S2). (F). Rosettogram for all high-affinity beads in the 15 Mbp, prepared using the alternative data set that gave the contact map in (D). Only pink binding beads that both bind red proteins and are in clusters are considered. As grey and light-green binding beads are not considered, and as these are often found in long runs, the effects of such long runs on the appearance of the rosettogram are minimized; choice of only pink beads that bind red proteins further minimizes the effects of runs of adjacent pink beads. Many red pixels abut in one row, indicating the formation of many rosettes involving nearest-neighbor pink beds. The f_d value is also low, indicating many local contacts and an ordered structure.

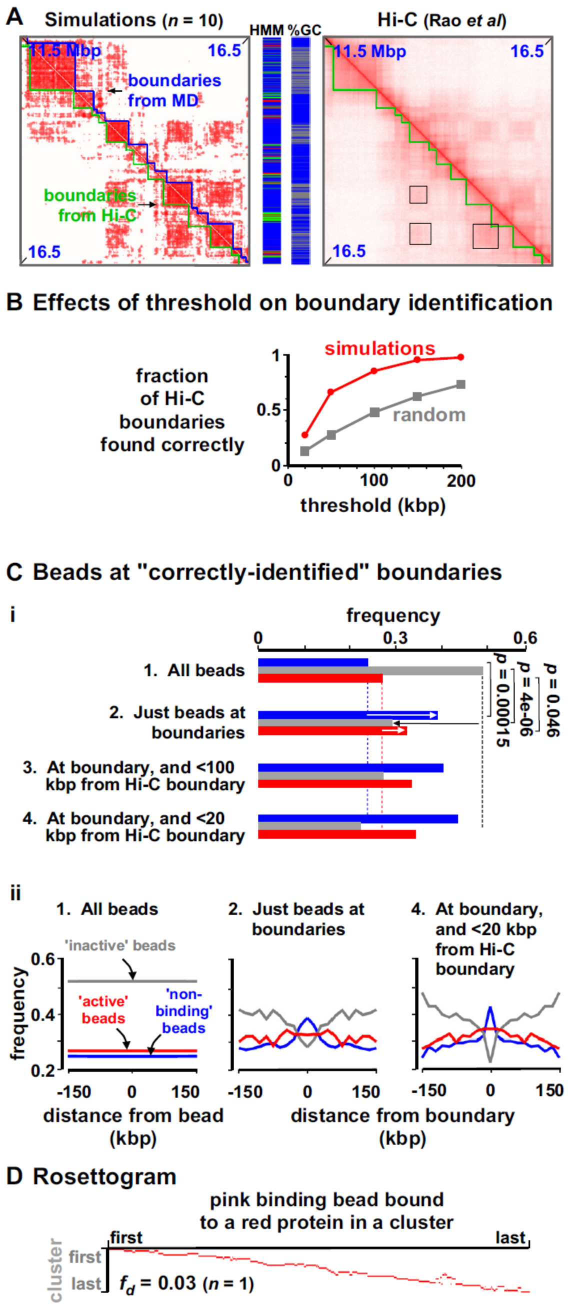
A Simulations (n = 10)
HMM %GC
Hi-C (Rao et al)
11.5 Mbp
16.5
boundaries from MD
boundaries from Hi-C
B Effects of threshold on boundary identification
fraction of Hi-C boundaries found correctly
simulations
random
threshold (kbp)
C Beads at "correctly-identified" boundaries
i
frequency
1. All beads
2. Just beads at boundaries
3. At boundary, and <100 kbp from Hi-C boundary
4. At boundary, and <20 kbp from Hi-C boundary
p = 0.00015
p = 4e-06
p = 0.046
ii
1. All beads
2. Just beads at boundaries
4. At boundary, and <20 kbp from Hi-C boundary
'inactive' beads
'active' beads
'non-binding' beads
frequency
distance from bead (kbp)
distance from boundary (kbp)
D Rosettogram
pink binding bead bound to a red protein in a cluster
first
last
cluster
f_d = 0.03 (n = 1)

Figure S13. Some properties of structures seen in simulations of chromosome 19 in GM12874 cells. Simulations were those used for Figure 5. (A) Zooms of contact maps reproduced from Figure 5C and D with added boundaries (21 and 20 kbp binning for data from simulations and Hi-C). Boundaries were determined using the "difference" plot aided by visual inspection (simulations – blue lines; Hi-C – green lines). Tracks between zooms (HMM states and %GC, colored as in Fig. 5A) show there is only partial correlation with domains in data from both simulations and Hi-C. Dashed rectangles in the Hi-C map mark (off-diagonal) blocks of distant contacts seen in both sets of data. (B) Effects of threshold on correct identification of boundaries (determined by visual inspection of the whole chromosome). A boundary is "correctly" predicted if it lies within a distance less than the threshold away from a boundary seen in Hi-C data. The grey line shows a control plot which gives the fraction of "correctly-determined" boundaries found by scattering randomly the same number of boundaries found in a simulation throughout the genomic region analysed. Error bars (±SD) in the random control are smaller than the square symbols and so cannot be seen, and the difference between points on the two curves are highly significant (typically $p < 10^{-6}$, see Table S2). (C) Correctly-identified boundaries in the whole chromosome are rich in active (pink and light-green) beads, and poor inactive (grey) beads. The frequencies of blue, grey, and pink+light-green beads (collectively depicted here by red bars and curves) in different sets of beads were calculated. Set 1: all beads. Set 2: Beads lying within 100 kbp of a boundary (identified manually as in Figure 5). Sets 3 and 4: The sub-sets of set 2 that also lie within 100 and 20 kbp of a boundary identified in Hi-C data. (i) Beads at boundaries are rich in active (pink+light-green) and blue beads, and depleted of inactive (grey) beads (arrows; p values assessed assuming Poisson distributions). (ii) The frequencies of different beads (in sets 1, 2 and 4) in the 150 kbp on each side of either each bead in set 1, or of boundaries in sets 2 and 4. Boundaries are rich in blue (blue curves) and active beads (red curves), and poor in inactive ones (grey curves). (D). Rosettogram for all high-affinity beads (pink) in the chromosome that both bind red proteins and are in clusters. Considering a sub-set of beads here has various advantages. First, as grey and light-green binding beads are not considered, and as these are often found in long runs, the effects of such long runs on the appearance of the rosettogram are minimized. Second, choice of only pink beads that bind red proteins further minimizes the effects of runs of adjacent pink beads. Third, these restrictions allow us to include all relevant beads in the whole chromosome in one plot. Many red pixels abut in one row, indicating the formation of many rosettes involving neighboring pink beads. The f_d is also very low, indicating an ordered structure.

IX. SUPPLEMENTARY TABLES

Row	Fig.	pattern	low aff. sites	f_d	n pixels	n clusters	% rosettes
1	1Avi	every 20	+	0.13 ± 0.02	173 ± 2.4	21 ± 0.8	0.37± 0.04
2	S2Aiv	every 20	-	0.2 ± 0.02	146 ± 3.6	20 ± 0.7	0.24 ± 0.03
3	S2Biv	random	+	0.06 ± 0.002	201 ± 1.4	17 ± 1.0	0.5± 0.03
4	S2Biv	random 20	-	0.12 ± 0.01	193 ± 3.1	20 ± 0.8	0.35± 0.07
5	2Aiv	alt. every 20	-	0.51 ± 0.02	92 ± 5.4	19 ± 1.0	0.18± 0.03
6	S4iv	alt. every 20	-	0.13 ± 0.01	186 ± 2.2	25 ± 0.8	0.38± 0.06

Supplementary Table S1: Some properties of rosettes found in different simulations (n= 5, except for the case in row 4 when n= 6). In the table: (i) f_d= fraction disorganized; (ii) Number of pixels: number of pixels in rosettogram; (iii) Number of clusters: number of clusters in rosettogram; (iv) alt.=alternating (pink and light green beads); (v) $\%\texttt{rosettes}$= percentage of rows with contiguous pixels.

row	thr (kbp)	p chr12	p chr12 (auto)	p chr6 (GC)	p chr6 (state)	p chr19
1	20	0.0011	0.01	0.35	0.61	2.6e-11
2	50	0.0024	0.003	0.017	0.14	<1e-11
3	100	0.000061	0.02	0.0011	0.13	< 1e-11
4	150	0.00048	0.37	0.000032	0.15	< 1e-11
5	200	0.0027	0.28	0.00015	0.045	4.6e-11

Supplementary Table S2: Comparison of boundaries seen in data from simulations and Hi-C. Data is from Figures 4 (chr12), S12 (chr6 using either %GC or HMM state 12 to identify grey beads) and 5 (chr19). Boundaries were identified by visual inspection, except for chr12, automated when boundaries were identified in an automated way (see Fig. S11B). In the table: thr=threshold; auto=automated; GC=using % GC; state=using only chromatin state track.

X. SUPPLEMENTARY MOVIE CAPTIONS

Movie S1: This Movie illustrates the simulation in Figure 1A. It can be seen that binding of red "transcription factors" creates loops and rosettes; the resulting clusters of red factors enlarge until they reach their steady-state size (i.e., they do not coarsen indefinitely).

Movie S2: Same as Movie S1, but now without chromatin to show the clustering of proteins more clearly.

Movie S3: This Movie illustrates the simulation in Figure 2A. It can be seen that the red and green factors form segregated clusters. Closer inspection shows that loops often involve non-nearest neighbor binding sites. As a result the disorganized fraction f_d in the rosettogram in Figure 2Aiv is high.

Movie S4: Same as Movie S3, but now without chromatin to show the segregation into clusters of separate red and green factors more clearly.

Movie S5: This Movie illustrates one simulation from Figure 4, and follows the dynamics of the domain formation in the simulated chr12:85000000-100000000 bp region in human chromosomes. Black proteins and red factors bind to hetero- and eu-chromatin, respectively. It can be seen that large heterochromatin domains form; their coarsening is arrested, or significantly slowed down, by the intervening (active) euchromatic domains.

Movie S6: This Movie shows a zoom on an internal region of the polymer simulated in Movie S5, to show details of the dynamics more clearly.

Movie S7: This Movie illustrates one simulation from Figure 5, and follows the evolution of domains. Black proteins and red factors bind to hetero- and eu-chromatin, respectively. Note the euchromatin on the surface of the large heterochromatic domains, and the slow (or arrested) coarsening of the black domains.

XI. REFERENCES

1. Brackley, C. A., Morozov, A. N. and Marenduzzo, D. Models for twistable elastic polymers in Brownian dynamics, and their implementation for LAMMPS. *J. Chem. Phys.* **140**, 135103 (2014).
2. Rosa, A., and Everaers, R. (2008). Structure and dynamics of interphase chromosomes. *PLoS Comput Biol* **4**, e1000153.
3. Rao, S. S. P., Huntley, M. H., Durand, N.C., Stamenova, E.K., Bochkov, I.D., Robinson, J.T., Sanborn, A. L., Machol, I., Omer, A. D., Lander, E. S. and Aiden, E. L. (2014). A 3D map of the human genome at kilobase resolution reveals principles of chromatin looping. *Cell* **159**, 1-16.
4. Dixon, J.R., Selvaraj, S., Yue, F., Kim, A., Li, Y., Shen, Y., Hu, M. Liu, J. S., and Ren, B. (2012). Topological domains in mammalian genomes identified by analysis of chromatin interactions. *Nature* **485**, 376-380.
5. Hsieh, T.-H. S., Weiner, A., Lajoie, B., Dekker, J., Friedman, N. and Rando, O. J. (2015). Mapping Nucleosome Resolution Chromosome Folding in Yeast by Micro-C. *Cell* **162**, 108-119.
